# The effect of morning vs evening exercise training on glycaemic control and serum metabolites in overweight/obese men: a randomised trial

**DOI:** 10.1007/s00125-021-05477-5

**Published:** 2021-05-19

**Authors:** Trine Moholdt, Evelyn B. Parr, Brooke L. Devlin, Julia Debik, Guro Giskeødegård, John A. Hawley

**Affiliations:** 1 Department of Circulation and Medical Imaging, Norwegian University of Science and Technology, Trondheim, Norway; 2 Women’s Clinic, St Olavs Hospital, Trondheim, Norway; 3 Exercise & Nutrition Research Program, Mary MacKillop Institute for Health Research, Australian Catholic University, Fitzroy, VIC Australia; 4 Department of Dietetics, Nutrition and Sport, La Trobe University, Melbourne, VIC Australia

**Keywords:** Blood glucose level, Circadian rhythm, High-fat diet, High-intensity interval training, Serum metabolomics

## Abstract

**Aims/hypothesis:**

We determined whether the time of day of exercise training (morning vs evening) would modulate the effects of consumption of a high-fat diet (HFD) on glycaemic control, whole-body health markers and serum metabolomics.

**Methods:**

In this three-armed parallel-group randomised trial undertaken at a university in Melbourne, Australia, overweight/obese men consumed an HFD (65% of energy from fat) for 11 consecutive days. Participants were recruited via social media and community advertisements. Eligibility criteria for participation were male sex, age 30–45 years, BMI 27.0–35.0 kg/m^2^ and sedentary lifestyle. The main exclusion criteria were known CVD or type 2 diabetes, taking prescription medications, and shift-work. After 5 days, participants were allocated using a computer random generator to either exercise in the morning (06:30 hours), exercise in the evening (18:30 hours) or no exercise for the subsequent 5 days. Participants and researchers were not blinded to group assignment. Changes in serum metabolites, circulating lipids, cardiorespiratory fitness, BP, and glycaemic control (from continuous glucose monitoring) were compared between groups.

**Results:**

Twenty-five participants were randomised (morning exercise *n* = 9; evening exercise *n* = 8; no exercise *n* = 8) and 24 participants completed the study and were included in analyses (*n* = 8 per group). Five days of HFD induced marked perturbations in serum metabolites related to lipid and amino acid metabolism. Exercise training had a smaller impact than the HFD on changes in circulating metabolites, and only exercise undertaken in the evening was able to partly reverse some of the HFD-induced changes in metabolomic profiles. Twenty-four-hour glucose concentrations were lower after 5 days of HFD compared with the participants’ habitual diet (5.3 ± 0.4 vs 5.6 ± 0.4 mmol/l, *p* = 0.001). There were no significant changes in 24 h glucose concentrations for either exercise group but lower nocturnal glucose levels were observed in participants who trained in the evening, compared with when they consumed the HFD alone (4.9 ± 0.4 vs 5.3 ± 0.3 mmol/l, *p* = 0.04). Compared with the no-exercise group, peak oxygen uptake improved after both morning (estimated effect 1.3 ml min^−1^ kg^−1^ [95% CI 0.5, 2.0], *p* = 0.003) and evening exercise (estimated effect 1.4 ml min^−1^ kg^−1^ [95% CI 0.6, 2.2], *p* = 0.001). Fasting blood glucose, insulin, cholesterol, triacylglycerol and LDL-cholesterol concentrations decreased only in participants allocated to evening exercise training. There were no unintended or adverse effects.

**Conclusions/interpretation:**

A short-term HFD in overweight/obese men induced substantial alterations in lipid- and amino acid-related serum metabolites. Improvements in cardiorespiratory fitness were similar regardless of the time of day of exercise training. However, improvements in glycaemic control and partial reversal of HFD-induced changes in metabolic profiles were only observed when participants exercise trained in the evening.

**Trial registration:**

anzctr.org.au registration no. ACTRN12617000304336.

**Funding:**

This study was funded by the Novo Nordisk Foundation (NNF14OC0011493).

**Graphical abstract:**

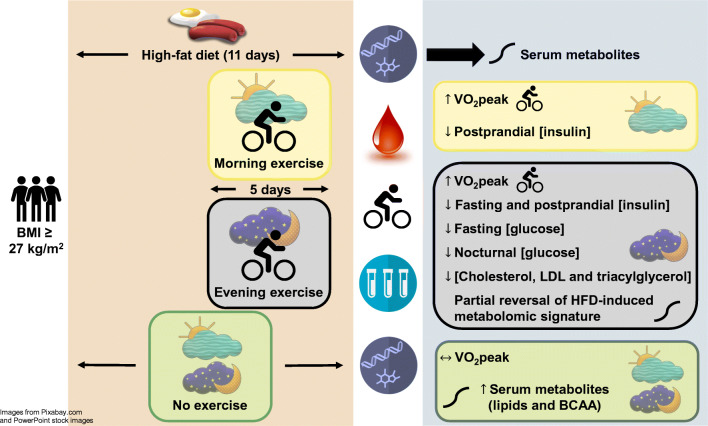

**Supplementary Information:**

The online version contains peer-reviewed but unedited supplementary material available at 10.1007/s00125-021-05477-5.

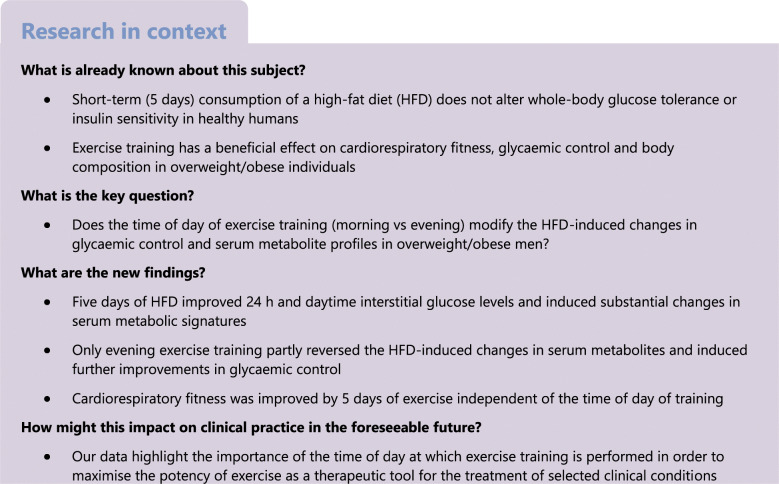



## Introduction

Several metabolic conditions, including obesity, impaired glucose tolerance and type 2 diabetes mellitus, stem from the adoption of a sedentary lifestyle and excess energy intake, with health guidelines advocating diet modification and exercise training as the first line of attack for the management of these disorders [1]. Diet recommendations emphasise food-based goals and a decrease in energy intake [2] while exercise guidelines encompass a range of activities to attain a prescribed ‘volume threshold’ to confer health benefits [3]. However, the time of day to exercise for optimal health benefits is currently unknown.

Recent investigations in rodents utilising gain-of-function/loss-of-function models and in humans employing omics technologies, have identified pathways that are differentially activated by exercise in a daytime-dependent manner [4]. In mice, the molecular circadian clock in peripheral tissues responds to the time of day of exercise, suggesting that skeletal muscle contraction relays important information for synchronisation of circadian clocks throughout the body [5]. Exercise also elicits distinct daytime muscle transcriptomic and metabolic signatures in mice and humans [4]. Indeed, timing is critical in amplifying the beneficial impact of exercise on metabolic pathways within muscle and systemic energy homeostasis [6]. Research exploring the effects of the timing of exercise on metabolic health outcomes in humans is sparse [7]. Savikj et al. [8] reported that 2 weeks of high-intensity training (HIT) performed three times weekly in the afternoon was more efficacious than morning HIT at improving glucose profiles in men with type 2 diabetes, while exercise training in the afternoon led to more pronounced metabolic adaptations compared with training in the morning in metabolically compromised adults [9]. In contrast, Teo et al. [10] reported similar benefits for a range of glycaemic measures after 12 weeks of either morning or evening exercise in individuals with and without type 2 diabetes. However, in these studies [8–10] neither the timing nor composition of meals was controlled, making it difficult to determine whether exercise timing or other factors underpinned the improvements in glucose profiles. Here we measured whole-body and blood biomarkers and used high-throughput serum metabolomics to determine the interactive effects of the consumption of a standardised high-fat diet (HFD) and daily exercise training undertaken either in the morning or evening on markers of cardiometabolic health and profiles of circulating metabolites in overweight/obese men. We explored how an HFD might induce specific metabolomic ‘signatures’ and whether these were altered according to the time of day at which exercise was undertaken. We also determined the modulating effect of short-term exercise training on HFD-induced changes in glycaemic control and selected markers of metabolic health.

## Methods

### Trial design and participants

We completed a parallel-groups randomised trial with participants allocated (1:1:1) to one of three groups: two groups who performed exercise training either in the morning (morning exercise [EXam] group) or in the evening (evening exercise [EXpm] group); and a third group who did not undertake any exercise training (control group [CON]) but consumed the same diet throughout the intervention as participants who exercised. Participants were recruited via social media and community advertisements. Eligibility criteria were as follows: male sex; aged 30–45 years; BMI 27.0–35.0 kg/m^2^; and sedentary lifestyle (<150 min/week moderate-intensity exercise for >3 months and sitting for >5 h each day). Potential participants were excluded if they met any of the following criteria: known CVD or type 2 diabetes; major chronic illness that impairs mobility or eating/digestion; taking prescription medications (i.e. β-blockers, anti-arrhythmic drugs, statins or insulin sensitising drugs); previous bariatric surgery; shift-work; smoking; strict dietary intake regimes (i.e. vegan, avoidances of principal study foods, not regularly consuming a breakfast meal, not regularly consuming three meals per day, actively trying to lose weight); or not being weight stable (±5 kg) for the last 3 months. Data were collected at the St Patrick’s (Fitzroy, VIC) campus of Australian Catholic University. The trial was approved by the Human Research Ethics Committee of the Australian Catholic University (2016-254H) and was registered with the Australian New Zealand Clinical Trials Registry (registration no. ACTRN12617000304336). Eligible participants received written information and provided informed consent before participation. The allocation sequence for participant randomisation was generated using a computer random number generator developed and administrated at the Unit for Applied Clinical Research at the Norwegian University of Science and Technology. The randomisation had varying block sizes, with the first, the smallest and the largest block defined by the computer technician. The researcher enrolling the participants (TM) received the allocation results onscreen and by email after registration of each new participant into the study and was unaware of the size of the blocks.

### Experimental protocol and interventions

**Fig. 1:**
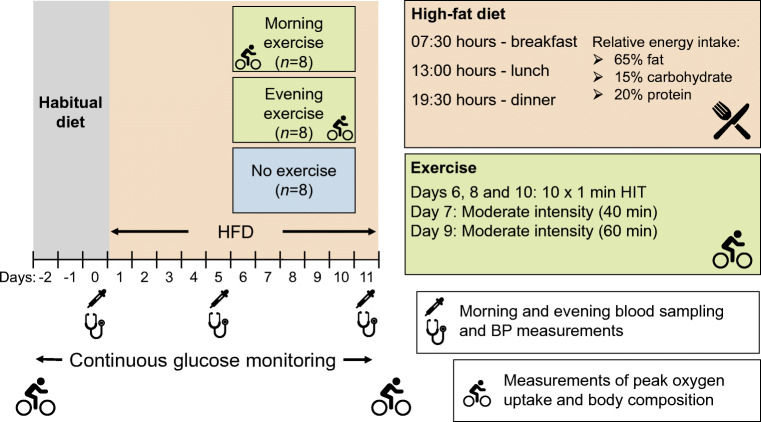
Overview of experimental design. The participants consumed a high-fat diet (HFD) for 11 days. Two groups of participants exercised, one group in the morning (*n* = 8) and the other in the evening (*n* = 8) on day 6–10, and one group of participants (*n* = 8) remained inactive for the whole study period. HIT, High-intensity interval training

Before commencement of dietary or exercise interventions, participants recorded their habitual intake of food and liquid in a food diary for three weekdays and one weekend day to allow for comparison with the macronutrient composition and energy intake of the study diet. Habitual dietary intake in the pre-intervention period was analysed using FoodWorks (Version 8; Xyris, QLD, Australia). The first 5 days of the investigation were the same for all participants and consisted of the introduction of an HFD while they remained sedentary (Fig. 1). For each of the 11 days of the HFD, participants received three pre-packed meals (breakfast, lunch and dinner), each containing 33.3% of the total daily energy intake. The macronutrient content of meals was 65% of energy from fat, 15% from carbohydrate and 20% from protein, with individualised total energy intake based on measurements of resting metabolic rate (detailed below). Participants were instructed to consume meals at prescribed times (07:30, 13:00 and 19:30 hours) and to abstain from alcohol. Water and coffee/tea (without sugar and milk) could be consumed ad libitum.

After the 5-day dietary harmonisation period, all participants consumed the HFD for a further 6 days at the prescribed times and with the same restrictions on liquids. On days 6–10, participants allocated to an exercise group performed once-daily exercise training at either 06:30 hours (EXam group) or 18:30 hours (EXpm group). Participants allocated to the control group (CON) did not perform any exercise but maintained their normal activities of daily living while remaining on the HFD (Fig. 1). Exercise training was performed on an electronically braked cycle ergometer (Lode Excalibur Sport, Lode, Groningen, the Netherlands). On days 6, 8 and 10, participants performed HIT (10 × 1 min work-bouts at 95–120% of individual peak power output (PPO), separated by 1 min low-intensity cycling). On days 7 and 9, participants in the exercise groups undertook a continuous bout of moderate-intensity cycling (60–65% of PPO) for 40 and 60 min, respectively. Participants allocated to an exercise training group received an extra 419 kJ snack (65% fat, 15% carbohydrate, 20% protein) following each exercise session.

### Assessments and outcomes

Prior to intervention, participants underwent the following procedures: (1) a dual-energy x-ray absorptiometry (DXA) (GE Lunar [USA] iDXA Pro, encore software Version 16) scan to estimate body composition; (2) measurement of resting energy expenditure (TrueOneRMR; Parvo Medic, Sandy, UT, USA) to estimate daily energy requirements; (3) girth measurements of waist and hip; (4) measurement of seated systolic and diastolic BP and resting heart rate using an automated oscillometric blood pressure monitor (Welch Allyn, NY, USA); and (5) a maximal cycling test with measurement of expired gases (Parvo Medic, Sandy, UT, USA) to determine peak oxygen uptake ($\dot{V}{\mathrm{O}}_{2\mathrm{peak}}$) and PPO. Apart from resting energy expenditure, all assessments were repeated after the intervention period, with the $\dot{V}{\mathrm{O}}_{2\mathrm{peak}}$ test and the DXA scan undertaken 48–72 h after participants had completed the 11 days of the HFD.

Blood samples (18 ml) were drawn using a single forearm venepuncture before breakfast and after dinner, at baseline (day 0), after 5 days on the HFD (day 5) and after a further 5 days of the HFD, either with daily exercise or no exercise (day 11). None of the participants exercised on day 11 (Fig. 1). Participants were fitted with a continuous glucose monitor (CGM) system (iPro2 with Enlite sensor; Medtronic, Dublin, Ireland) placed on the lower back adipose site, and worn throughout the study. We calculated hourly values and defined daytime glucose as the mean glucose concentration from 06:00 hours to 22:00 hours and nocturnal glucose as the mean glucose concentration from 22:00 hours to 06:00 hours.

Participants wore an activPAL inclinometer (activPAL3^TM^tri-axial physical activity monitor; PAL-technologies, Glasgow, UK) on the thigh and an ActiGraph accelerometer (ActiGraph GTX3+; Pensacola, FL, USA; during waking hours only) around the waist to estimate physical activity and movement patterns. Additionally, a SenseWear armband (Bodymedia, Pittsburgh, PA, USA) was worn on the upper arm for estimates of energy expenditure.

### Biochemical analyses

Total cholesterol, HDL-cholesterol, LDL-cholesterol and triacylglycerol concentrations were analysed in whole blood using Cobas b 101 (Roche Diagnostics, Basel, Switzerland) and plasma glucose concentration using the hexokinase methods with a YSI 2900 STAT Plus (YSI Life Sciences, Yellow Springs, OH, USA). Serum insulin concentrations were determined in duplicate using ELISA (ALPCO, Salem, NH, USA). HOMA-IR was calculated according to the following formula: fasting serum insulin (μU/ml) × fasting plasma glucose (mmol/l) / 22.5 [11].

### Serum metabolomics

Metabolomics analysis was undertaken by Metabolon (Durham, NC, USA) [12]. Thawed serum was methanol extracted and analysed using ultra-HPLC–tandem MS (UPLC-MS/MS) positive mode, UPLC-MS/MS negative mode and GC-MS. The UPLC-MS/MS platform used a Waters Acquity UPLC with Waters UPLC BEH C18–2.1× 100 mm, 1.7 mm columns and a ThermoFisher LTQ MS, including an electrospray ionisation source and a linear ion-trap mass analyser. Samples for GC-MS analysis were dried under vacuum desiccation for a minimum of 18 h prior to being derivatised using bis(trimethylsilyl) trifluoroacetamide. Using helium as carrier gas and at a temperature ramp from 60 °C to 340 °C within a 17 min period, derivatised samples were separated on a 5% phenyldimethyl silicone column. Samples were analysed on a Thermo-Finnigan Trace DSQ fast-scanning single-quadrupole MS operated at unit mass resolving power with electron impact ionisation and a 50e750 atomic mass unit scan range. Metabolites were identified by automated comparison of the ion features in the experimental samples to a reference library of chemical standard entries. The reference library included retention time, molecular mass (m/z), preferred adducts and in-source fragments, as well as associated MS spectra, and were curated by visual inspection for quality control using software developed at Metabolon [13].

### Statistical methods

A formal sample size calculation was not undertaken due to the exploratory nature of the research question. Data are expressed as means (±SD) and estimates with 95% CIs. We used paired *t* tests to assess differences in traditional biomarkers of cardiometabolic health between baseline and after 5 days of HFD, for the entire cohort. To determine between-group differences in traditional biomarkers we used mixed models with participant as random effect, and with two dummy variables to uniquely identify the two exercise groups (EXam and EXpm) and their interactions with time (categorical) as fixed effects. We assumed no systematic differences between groups at baseline or after 5 days of HFD. The analyses were repeated to test for differences between the EXam and EXpm groups. We adjusted for baseline values for each outcome variable as recommended by Twisk et al. [14]. For the traditional clinical biomarkers, *p* values <0.05 were considered statistically significant (without any adjustment for multiple comparisons).

#### Metabolomics analysis

The raw metabolomic data consisted of 897 quantified metabolites. For the data analysis, the original scale was used, with values normalised in terms of raw area counts by Metabolon. Metabolites with >30% missing values (*n* = 105, of which 93 were xenobiotics) were removed prior to analysis, as was lidocaine, resulting in 791 metabolites. Values below the limit of quantification were imputed with a value equal to half of the lowest detected value of the corresponding metabolite. Principal component analysis (PCA) was undertaken on serum metabolites to assess the presence of a systematic trend in the change of the metabolic profiles of the participants during the harmonisation period (habitual diet vs HFD), and to assess differences in metabolic profiles induced by exercise training. PCA was performed using PLS_Toolbox 8.7.2 (Eigenvector Research, Wenatchee, WA, USA) in Matlab R2017b. Percentage changes of the metabolite concentrations as induced by the HFD were calculated. The significance of the change between habitual diet and HFD was determined by paired *t* tests. Mixed models were used to test between-group differences while adjusting for baseline values (metabolite values at day 5, prior to the exercise intervention), with participant as random effect, time and time × group interactions as fixed effects, and metabolite concentration as the dependent variable. Separate models were built for comparison of EXam vs CON, EXpm vs CON, and EXam vs EXpm. The *p* values were adjusted using the Benjamini–Hochberg procedure [15] and significance was considered for *q* values <0.05. Analyses were performed on the morning and evening samples separately.

Heatmaps within each class of metabolites (lipids, amino acids, carbohydrates, peptides, co-factors and vitamins, energy, nucleotides, and xenobiotics) were ordered by hierarchical clustering using Spearman correlation as a similarity measure and average linkage as a distance measure. Clustering was performed on morning samples, and the same metabolite order was used for evening samples.

## Results

### Participants

**Fig. 2:**
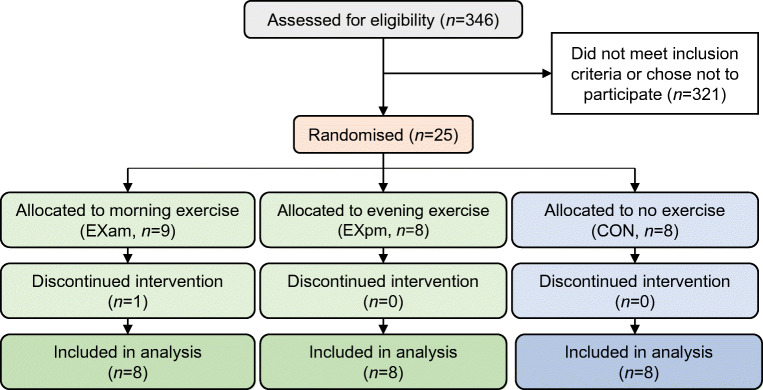
Consolidated Standards of Reporting Trials (CONSORT) flow diagram

Twenty-five participants were randomised and 24 completed the full protocol (Fig. 2). Table 1 shows the baseline characteristics of participants. Recruitment commenced in March 2017, with the last follow-up in August 2017. The interventions produced no unintended or adverse effects.

**Table 1:** Baseline characteristics of participants included in analyses

Characteristic	EXam (*n* = 8)	EXpm (*n* = 8)	CON (*n* = 8)
Age (years)	35 ± 4	36 ± 5	36 ± 4
Body mass (kg)	103.1 ± 16.1	99.3 ± 8.1	94.5 ± 10.4
BMI (kg/m^2^)	31.9 ± 1.9	31.0 ± 2.8	30.7 ± 2.3
Fat mass (kg)	36.1 ± 7.0	32.9 ± 5.3	31.3 ± 3.0
Fat mass (%)	36.0 ± 3.1	34.1 ± 3.4	33.6 ± 3.6
Fat-free mass (kg)	67.2 ± 10.1	66.7 ± 3.9	65.4 ± 9.0
Visceral fat mass (g)	2013 ± 1424	1480 ± 573	1969 ± 495
Systolic BP (mmHg)	126 ± 10	131 ± 9	129 ± 8.6
Diastolic BP (mmHg)	81 ± 5	84 ± 7	85 ± 8.6
$\dot{V}{\mathrm{O}}_{2\mathrm{peak}}$ (ml kg^−1^ min^−1^)	28.5 ± 6.1	31.2 ± 4.4	29.0 ± 4.3
PPO (W)	209 ± 63	208 ± 26	193 ± 33
Glucose (mmol/l)	4.9 ± 0.5	4.8 ± 0.5	5.0 ± 0.4
Insulin (pmol/l)	69 ± 44	46 ± 35	63 ± 35
HOMA-IR	2.6 ± 1.8	1.7 ± 1.3	2.4 ± 1.5
Cholesterol (mmol/l)	5.0 ± 0.8	4.3 ± 0.6	4.9 ± 0.8
HDL-cholesterol (mmol/l)	1.01 ± 0.2	1.10 ± 0.2	0.99 ± 0.2
LDL-cholesterol (mmol/l)	3.3 ± 0.6	2.7 ± 0.5	3.1 ± 0.8
Triacylglycerol (mmol/l)	1.63 ± 0.7	1.23 ± 0.6	1.78 ± 0.8

Data are means ± SD

### Habitual diet, physical activity and sleep

The contribution to total energy intake in the participants’ habitual diet was as follows: fat 36 ± 4%; carbohydrates 45 ± 9% and protein 18 ± 3%; the diet also contained alcohol (1%) and dietary fibre (2%). Before the trial, participants were physically active for 182 ± 66 min/day of light intensity (1.5–2.9 metabolic equivalent of task [MET]), 96 ± 42 min/day of moderate-intensity (3.0–5.9 MET) and 1 ± 2 min/day of vigorous intensity (≥6.0 MET) exercise, taking 7547 ± 3253 steps/day. Levels of physical activity did not change during the first 5 days, when all participants consumed the HFD, nor did the time spent in bed (estimated from ActivPAL inclinometer data) when compared with the subsequent 5 days of HFD with or without exercise (electronic supplementary material [ESM] Table 1). Seven participants in EXam shifted their wake-up times (by 1–2 h) and six of these compensated by going to bed 1–2 h earlier.

### Effects of 5 days of HFD

**Fig. 3:**
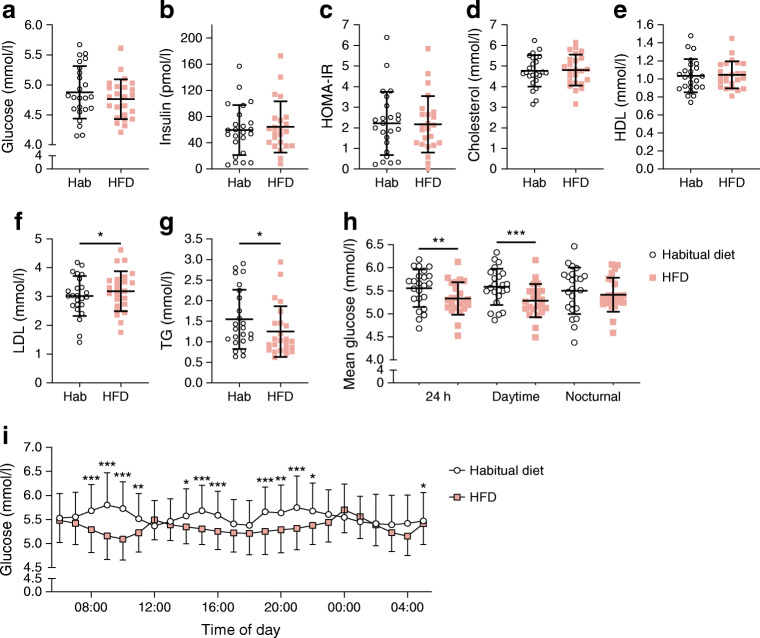
(**a**–**g**) Biomarkers measured in the fasted state in participants before (habitual diet) and after 5 days of high-fat diet (HFD): plasma glucose (**a**); serum insulin (**b**); HOMA-IR (**c**); blood cholesterol (**d**), HDL-cholesterol (**e**), LDL-cholesterol (**f**) and triacylglycerol (**g**). The plots depict means ± SD with individual data points. (**h**) CGM-based plasma glucose concentrations for 24 h, daytime, and nocturnal glucose in participants before (habitual diet) and after 5 days of HFD. The plot depicts means ± SD with individual data points. (**i**) Hourly glucose concentrations (mean ± SD) in participants before (habitual diet) and after 5 days of HFD. *p < 0.05, **p < 0.01 and ***p < 0.001 for HFD vs habitual diet. Hab, habitual diet; HDL, HDL-cholesterol; LDL, LDL-cholesterol; TG, triacylglycerol

Fasting triacylglycerol decreased from 1.54 ± 0.7 to 1.25 ± 0.6 mmol/l (*p* = 0.03) and fasting LDL-cholesterol increased from 3.0 ± 0.7 to 3.2 ± 0.7 mmol/l (*p* = 0.049) after 5 days of HFD (Fig. 3). Measures of insulin sensitivity (fasting and postprandial plasma glucose, fasting and postprandial serum insulin and HOMA-IR) were unchanged during this period, nor were there any changes in total cholesterol, HDL-cholesterol, or BP (ESM Table 2). The HFD decreased CGM-based 24 h glucose concentrations (from 5.6 ± 0.4 to 5.3 ± 0.4 mmol/l, *p* = 0.001), mainly due to lower daytime glucose concentrations (Fig. 3).

#### Serum metabolites

**Fig. 4:**
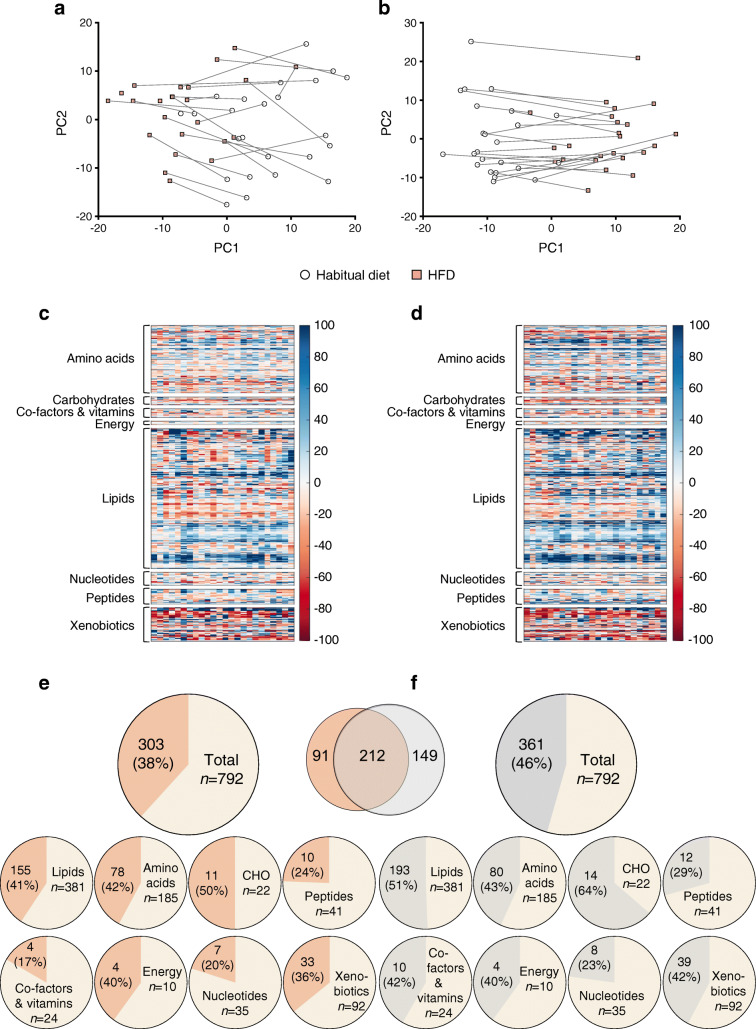
Serum metabolites before and after 5 days of high-fat diet (HFD). (**a**, **b**) PCA of serum metabolites measured in morning (**a**) and evening (**b**) samples. (**c**, **d**) Heatmaps of changes in metabolites in response to HFD in morning (**c**) and evening (**d**) samples, with the same clustering order of metabolites in both samples. For improved visualisation, the colour bars are limited to −100% to +100%. (**e**, **f**) Number of metabolites that changed in morning (**e**) and evening (**f**) samples, categorised according to metabolic class. The Venn diagram shows the overlap between morning and evening samples. PC, principal component

**Fig. 5:**
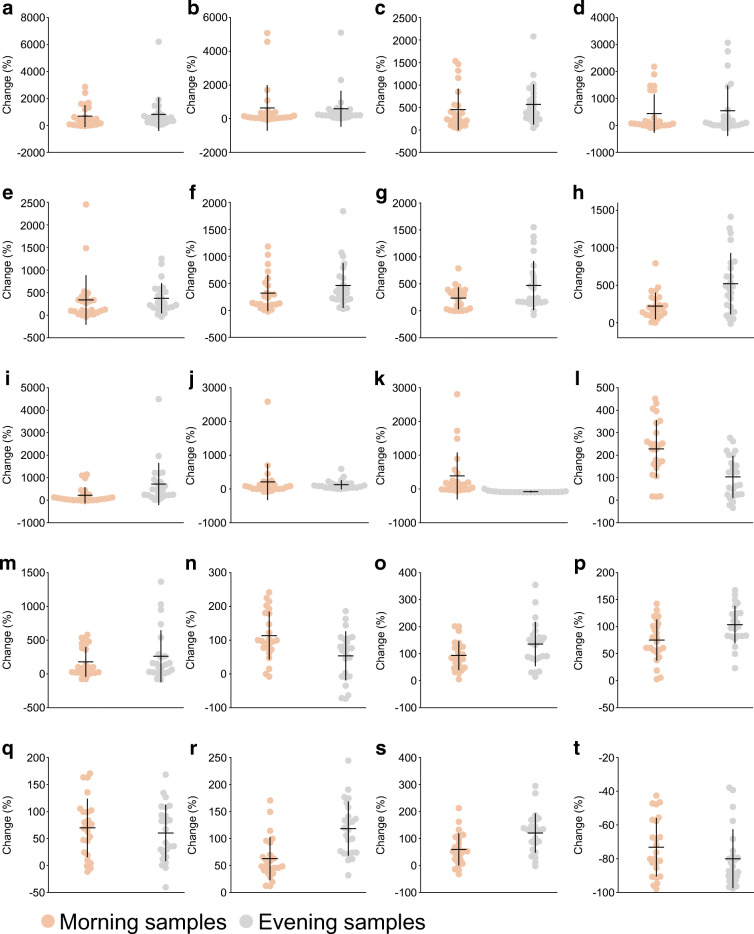
Top ten lipid and amino acid metabolites that changed significantly (all *q* < 0.05) in the morning samples after 5 days of high-fat diet (HFD) compared with the participant’s habitual diet. (**a**–**j**) Relative change (%) in the lipid metabolites 12,13 DiHOME (**a**), pristanate (**b**), 3-hydroxybutyrylcarnitine (1) (**c**), *N*-stearoyl-sphingadienine (d18:2/18:0) (**d**), acetoacetate (**e**), 4-methylhexanoylglutamine (**f**), 3-hydroxysebacate (**g**), βOHB (**h**), suberoylcarnitine (C8-DC) (**i**) and glyco-β-muricholate (**j**). (**k**–**t**) Relative change (%) in the amino acid metabolites *S*-methylmethionine (**k**), methylcysteine sulfoxide (**l**), α-ketobutyrate (**m**), *S*-methylcysteine (**n**), 2-hydroxybutyrate/2-hydroxyisobutyrate (**o**), 2-aminobutyrate (**p**), *N*-acetylglycine (**q**), 3-hydroxy-2-ethylpropionate (**r**), 3-hydroxyisobutyrate (**s**) and 4-methoxyphenol sulfate (**t**). The plots depict means ± SD with individual data points

PCA of serum metabolites revealed systematic changes in metabolism between the habitual diet and the HDF. Of 792 metabolites, 303 were altered in the morning samples and 361 were altered in the evening samples (Fig. 4). There were significant changes in all classes of metabolites. We highlight some of the prominent changes in the morning (fasting) samples only, which are reported with adjusted *p* values (*q* values). The HFD induced several distinct changes in fatty acid metabolism, with 155 of 381 lipid metabolites altered (Fig. 4). There were diet-induced increases in NEFA, including long-chain fatty acids (e.g. 10-nonadecenoate [19:1n9], +42%, *q* = 0.015 and oleate/vaccenate [18:1], +34%, *q* = 0.014) and dicarboxylate fatty acids (e.g. heptenedioate [C7:1-DC], +206%, *q* = 0.0003). There were increases in circulating acetylcarnitine (+54%, *q* < 0.0001) and several carnitine-conjugated fatty acids, and substantial elevations in the ketone bodies β-hydroxybutyrate (βOHB) (+224%, *q* = 0.0001) and acetoacetate (+ 340%, *q* = 0.0004). Sphingolipids as a class were significantly increased following the HFD (e.g. sphingomyelin [d18:0/18:0. D19:0/17:0], +98%, *q* = <0.0001) (ESM Table 3). The HFD altered 78 out of 185 amino acid metabolites in the morning samples, including elevations in metabolites related to branched-chain amino acid (BCAA) metabolism (e.g. 3-hydroxy-2-ethylpropionate,+63%, *q* < 0.0001; and 3-hydroxyisobutyrate,+60%, *q* = 0.001) (Fig. 5). Valine, leucine, and isoleucine all increased (*q* < 0.05). ESM Table 3 and ESM Table 4 show metabolites that changed in the morning and evening samples, respectively, following 5 days of HFD.

### Effects of exercise training

**Fig. 6:**
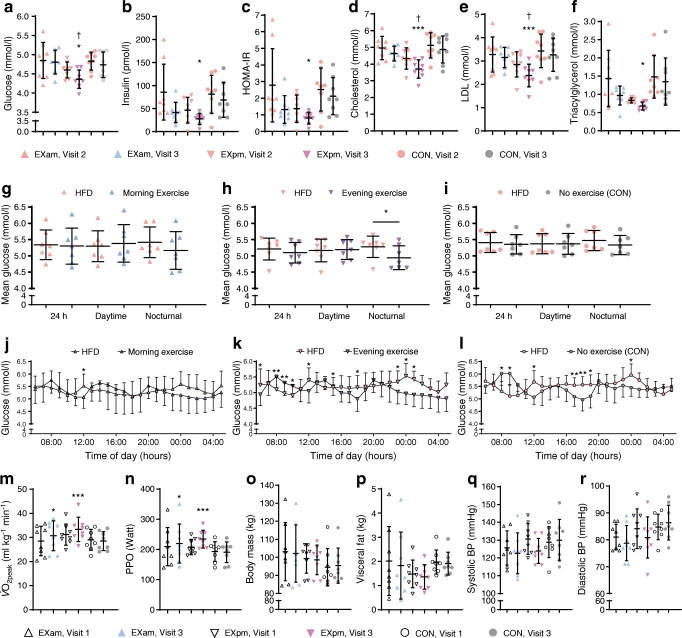
(**a**–**f**) Biomarkers measured in the fasted state in participants after 5 days of high-fat diet (HFD) (visit 2) and after exercise in the morning (EXam), exercise in the evening (EXpm) or no exercise (CON) after the intervention period (visit 3): plasma glucose (**a**); serum insulin (**b**); HOMA-IR (**c**); and blood cholesterol (**d**), LDL-cholesterol (**e**) and triacylglycerol (**f**). The plots depict means ± SD with individual data points. Comparison between groups are changes at visit 3, adjusted for visit 2 values. *p < 0.05 and ***p ≤ 0.001 vs CON; ^†^*p* < 0.05 vs EXam . (**g**–**i**) CGM-based glucose concentrations for 24 h, daytime and nocturnal glucose in the EXam (**g**), EXpm (**h**) and no exercise (**i**) groups. The plots depict means ± SD with individual data points. Changes are within groups. *p < 0.05. (**j**–**l**) Hourly glucose concentrations in the EXam (**j**), EXpm (**k**) and no exercise (**l**) groups. Data are mean ± SD and changes are within group. *p < 0.05 and **p < 0.01. (**m**–**r**) $\dot{V}{\mathrm{O}}_{2\mathrm{peak}}$ (**m**), PPO (**n**), body mass (**o**), visceral fat mass (**p**) and BP (**q**, **r**) at baseline (visit 1) and after the intervention (visit 3), according to group. The plots depict means ± SD with individual data points. Comparison between groups are changes at visit 3, adjusted for visit 1 values. *p < 0.05 and ***p ≤ 0.001 vs CON

Figure 6 displays measures for $\dot{V}{\mathrm{O}}_{2\mathrm{peak}}$, body composition and BP at baseline and post-intervention. Participants randomised to either EXam or EXpm completed all prescribed sessions. During HIT, the mean power output was 106 ± 5% of PPO (92 ± 4% of maximum heart rate). During the continuous exercise sessions, power output was 65 ± 2% and 60 ± 1% of PPO for the 40 min and 60 min session, respectively. $\dot{V}{\mathrm{O}}_{2\mathrm{peak}}$ and PPO increased for both exercise groups (Fig. 6 and ESM Table 5). Compared with CON, $\dot{V}{\mathrm{O}}_{2\mathrm{peak}}$ improved in the EXam group by 1.3 ml min^−1^ kg^−1^ (95% CI 0.5, 2.0, *p* = 0.003) and in the EXpm group by 1.4 ml min^−1^ kg^−1^ (95% CI 0.6, 2.2, *p* = 0.001). There were no between-group differences for BP or body composition (Fig. 6 and ESM Table 5).

There were between-group differences for changes in fasted circulating markers of metabolic health after the exercise intervention compared with after 5 days of HFD (Fig. 6 and ESM Tables 6, 7). Blood glucose, cholesterol and LDL-cholesterol decreased by a greater magnitude in the EXpm group compared with either CON or the EXam group, while insulin and triacylglycerols fell more in EXpm vs CON. In the evening samples (i.e. postprandial), circulating insulin decreased after both EXam and EXpm compared with no exercise, whereas cholesterol, triacylglycerol, and LDL-cholesterol only decreased after EXpm (ESM Tables 6, 7). Neither exercise group showed a decrease in their CGM-based 24 h glucose concentrations but individuals in the EXpm group had lower nocturnal glucose concentrations on the days they exercised (day 6–10) when compared with concentrations after the HFD only (4.9 ± 0.4 vs 5.3 ± 0.3 mmol/l, *p* = 0.04, Fig. 6).

#### Serum metabolites

**Fig. 7:**
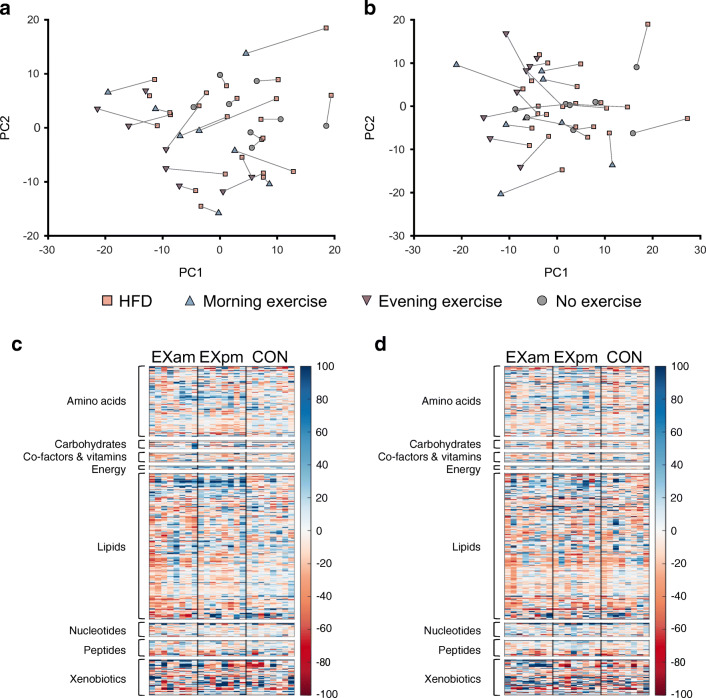
Serum metabolites before (visit 2) and after (visit 3) exercise in the morning (EXam), exercise in the evening (EXpm) or no exercise (CON). (**a**) PCA of serum metabolites measured in morning samples, according to group. (**b**) PCA of serum metabolites measured in evening samples, according to group. (**c**) Heatmaps of changes in metabolites in response to exercise in EXam, EXpm or CON groups, in morning samples. (**d**) Heatmaps of changes in metabolites in response to exercise in EXam, EXpm or CON groups, in evening samples, with the same clustering order of metabolites as in the morning samples. For improved visualisation, the colour bars are limited to −100% to +100%. PC, principal component

**Fig. 8:**
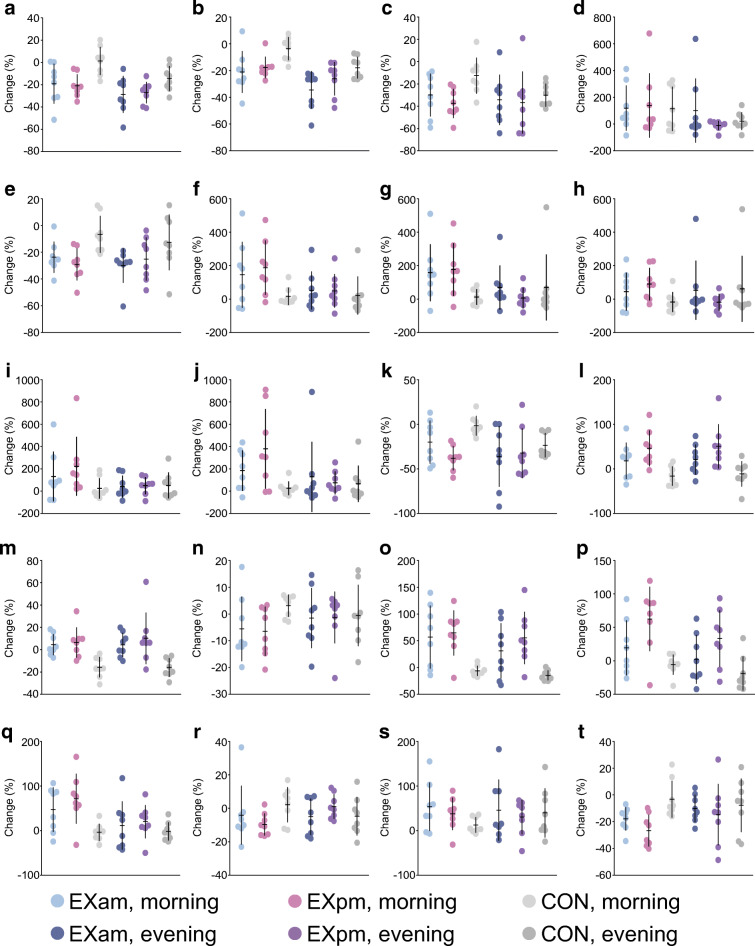
Relative change in lipid and amino acid metabolites that were significantly changed from habitual diet to high-fat diet (HFD) and that showed differential change in morning samples from the evening exercise (EXpm) group compared with no exercise (CON) (all *q* < 0.05), using mixed linear models. (**a**–**m**) Relative change (%) in the lipid metabolites sphingomyelin (d17:1/14:0, d16:1/15:0) (**a**), myristoyl dihydrosphingomyelin (d18:0/14:0) (**b**), 1-myristoyl-2-arachidonoyl-GPC (14:0/20:4) (**c**), hexanoylglycine (**d**), propionylcarnitine (C3) (**e**), 3-hydroxybutyrylcarnitine (1) (**f**), 3-hydroxybutyroylglycine (**g**), 3-hydroxysebacate (**h**), acetoacetate (**i**), βOHB (**j**), 1-palmitoleoyl-2-linolenoyl-GPC (16:1/18:3) (**k**), 5α-androstan-3β,17β-diol monosulfate (2) (**l**) and androstenediol (3β,17β) disulfate (1) (**m**). (**n**–**t**) Relative change (%) in the amino acid metabolites tryptophan (**n**), α-hydroxyisovalerate (**o**), 2-hydroxy-3-methylvalerate (**p**), 2-hydroxybutyrate/2-hydroxyisobutyrate (**q**), tyrosine (**r**), *N*-acetylglycine (**s**) and creatine (**t**). Values are means ± SD

Changes in serum metabolites from pre- to post-exercise training are displayed in Figs 7, 8. The changes in metabolites after the exercise interventions were less pronounced than those seen after 5 days of the HFD. PCA revealed smaller variation in metabolites between time points in CON compared with either of the exercise groups, although no differences for individual metabolites were observed between EXam and CON. For the EXpm group, we detected between-group differences for 56 metabolites in the morning samples and seven metabolites in the evening samples when compared with CON (ESM Tables 8, 9). The most profound differences between groups were for adrenal steroid metabolism, in which a reversal of the effect of the HFD was evident after exercise training. A total of 33 lipid metabolites were differentially altered after EXpm vs CON. Of the metabolites altered by the HFD, some changed differently between the EXpm group and CON. These were metabolites involved in fatty acid and amino acid metabolism, including ketone bodies, metabolites implicated in tryptophan metabolism, BCAA metabolism, tyrosine metabolism and glutathione metabolism (Fig. 8). Evening exercise partly reversed the effect of HFD-induced increase in sphingolipid synthesis, as evident by significant decreases in sphingomyelin (d17:1/14:0, d16:1/15:0) and myristoyl dihydrosphingomyelin (d18:0/14:0) in EXpm vs CON (Fig. 8, ESM Table 8).

## Discussion

In this parallel-groups randomised trial, we measured whole-body and blood biomarkers of metabolic health and used high-throughput metabolomics to explore the responses of overweight/obese men to a standardised nutritional challenge and time of day of exercise training. We report that compared with a sedentary control group, men who exercised in the evening but not the morning improved their fasting circulating glucose and insulin concentrations as well as LDL-cholesterol and triacylglycerol profiles. Furthermore, nocturnal glucose profiles were only improved in men who exercised in the evening. Notwithstanding time of day effects of exercise on traditional biomarkers of health, both morning and evening exercise improved cardiorespiratory fitness and systemic circulating insulin levels after standardised meals. The HFD induced substantial perturbations in circulating metabolites, while daily exercise had a smaller effect on serum metabolic profiles. Only the evening exercise group showed differential responses in metabolites compared with the no-exercise control group, with no significant differences between the morning and evening exercise groups.

We have previously reported that in overweight/obese men consumption of an HFD compared with a carbohydrate-rich diet robustly increased the number of metabolites related to lipid metabolism, independent of the time of day (i.e. morning vs evening) [16]. In the present study, short-term consumption of an HFD increased levels of circulating fatty acids, particularly circulating acetylcarnitine and carnitine-conjugated fatty acids. These findings suggest alterations in fat handling, specifically fatty acid β-oxidation, as conjugation of fatty acids with carnitine is required for transport across mitochondrial membranes and subsequent β-oxidation [17]. However, circulating metabolites are often intermediary or end products of different metabolic pathways and do not provide information regarding the turnover of molecules through a metabolism pathway.

Sphingolipid metabolism was substantially altered after just 5 days of an HFD, consistent with previous observations in rodents [18]. In addition to their roles in maintaining membrane structure and integrity, sphingolipids are bioactive lipids that promote arteriosclerosis and CVD [19]. Indeed, HFD-stimulated sphingolipid generation contributes to systemic insulin resistance, dysregulated lipid accumulation, and cytokine expression and secretion in skeletal muscle and adipose tissues, thus exacerbating pre-existing obesity-related disorders [20]. We show that exercise in the evening mitigated the HFD-induced increase in sphingolipid metabolism by lowering the levels of selected sphingomyelins, ceramides and myristoyl dihydrosphingomyelin. Ceramides are the precursor for sphingolipids and a major contributing factor in insulin resistance and CVD [21]. Previous work demonstrates that peak levels of glycerophospholipids and sphingolipids correlate with peak *BMAL1* (also known as *ARNTL*) expression, and that skeletal muscle taken from healthy humans exhibits a day–night rhythm in lipid metabolism [22]. These results suggest a potential role for exercise timing and skeletal muscle clocks in the regulation of lipid metabolism [23].

Ketone bodies are small lipid-derived molecules that serve as a circulating energy source for bioenergetic tissues in times of fasting and/or low carbohydrate availability [24] as well as signalling intermediates linking the outside environment to epigenetic gene regulation and cellular function [25]. Elevated blood ketone concentrations occur within 2–4 days of an HFD [24, 25]. Indeed, we observed rapid HFD-induced increases in ketone metabolism (Fig. 5), with the levels of acetoacetate and βOHB further augmented by evening exercise. Smaller changes were seen after exercise undertaken in the morning, but these were not different from those induced by the HFD alone. A growing area of scientific interest is the anabolic and anticatabolic properties of ketone bodies in skeletal muscle provided by therapeutic ketosis, as well as the effects of acetoacetate and βOHB on metabolic processes such as attenuation of glycolysis, hepatic glucose output and adipose tissue lipolysis [26]. Accordingly, identifying the mechanisms underlying diet- and/or exercise-induced changes in circulating metabolites such as acetoacetate and βOHB may provide targets that help regulate insulin sensitivity and/or discovery of novel roles for known metabolites.

Elevated levels of several serum amino acids are associated with obesity and insulin resistance [27]. Specifically, increases in leucine, isoleucine and valine have been linked to a number of chronic disorders including obesity, insulin resistance and type 2 diabetes (see [28]). Increased levels of BCAAs may serve as a biomarker for the early detection of insulin resistance and related conditions [29]. Despite the brevity of our HFD intervention, we observed increases in the levels of leucine, isoleucine and valine, with a concomitant reduction in circulating glycine and glutamine, the latter being inversely associated with type 2 diabetes risk [30]. Short-term exercise training was unable to reverse the HFD-induced changes in the profile of these amino acids. However, after evening exercise there were differential changes in the levels of circulating α-hydroxyisovalerate and 2-hydroxy-3-methylvalerate, metabolites involved in leucine, isoleucine, and valine metabolism, as well as in *N*-acetylglycine.

Studies in animals show that the time of day of physical activity can amplify the beneficial impact of exercise on metabolic pathways within skeletal muscle and on systemic energy homeostasis [6], although caution should be taken when extrapolating the results from investigations undertaken in mice to human clinical populations [31]. Our findings of exercise-induced improvements in glycaemic control in participants who exercised in the evening rather than the morning extend previous observations in individuals with type 2 diabetes [8] and people at risk of, or diagnosed with, type 2 diabetes [9]. Savikj et al. [8] reported that HIT undertaken in the morning increased glucose concentration acutely as well as on a subsequent rest day during a 2 week training intervention. Mancilla et al. [9] found that compared with participants who trained in the morning (a combination of aerobic and resistance-based exercises), participants who undertook the same training in the afternoon had better peripheral insulin sensitivity, insulin-mediated suppression of adipose tissue lipolysis, and fasting plasma glucose levels. However, glycaemic control and postprandial responses were similarly improved after 12 weeks of combined endurance and resistance exercise training undertaken in the morning vs evening in men and women with and without type 2 diabetes [10]. Long-term training studies incorporating early vs late training interventions are urgently needed to interrogate the effects of time of day specific exercise on metabolism and health outcomes.

In contrast to previous short-term training studies reporting no increase in cardiorespiratory fitness [32–34], we observed significant improvements in $\dot{V}{\mathrm{O}}_{2\mathrm{peak}}$ in both exercise groups after only 5 days of training, suggesting that the time of day of exercise did not influence gains in aerobic fitness. Human skeletal muscle mitochondrial oxidative capacity is highest towards the end of the day [35], coinciding with the timing of peak physical performance [36], with afternoon exercise being more efficacious than morning exercise at improving glucose profiles in both the current study and previous reports [8, 9]. Synchrony between skeletal muscle and other insulin-sensitive tissues is critical to maintain whole-body glucose homeostasis, with insulin secretion and sensitivity under circadian regulation, peaking early in the day and declining in the late afternoon/evening in healthy individuals [37]. The evening decrease in glucose tolerance is due to a decrease in both insulin sensitivity and beta cell function [38]. In individuals with type 2 diabetes, however, this circadian pattern in glycaemic control is blunted [38]. It should be noted that many of the studies examining the variability of glucose metabolism have been performed in the absence of an exercise challenge [37] and therefore our finding that nocturnal glucose profiles were only improved in men who trained in the evening has important implications for exercise prescription in individuals who are metabolically challenged.

Strengths of the current study include its randomised design, with a sedentary control group, and adequate statistical power to permit inferences regarding the effects of morning or evening exercise in overweight/obese men consuming an HFD. Our rigorous dietary control in which participants were provided with all meals and prescribed times for eating allowed us to investigate the biological outcomes of our diet and exercise protocols with little uncontrolled environmental stimuli. In addition to traditional biomarkers of health status, we undertook comprehensive metabolic profiling of serum samples to identify metabolomic signatures resulting from our interventions. Differences in the timing of biological sampling since the last training session (12 vs 24 h) is a limitation in our study. This discrepancy is inherent in any investigation of the time of day of exercise, with no clear solution other than comprehensive time course studies involving multiple tissue sampling [39]. Exercising early in the morning could induce sleep-loss-related insulin resistance [40], and our study participants had to wake up early to train. Despite the fact that we observed no difference in the estimated time spent in bed for either group between the HFD-only period and the days participants exercised, circadian misalignment may be a confounding factor when interpreting our results, although exercise mitigates sleep-loss-induced changes in glucose tolerance [41]. Another limitation is the inclusion of men only, meaning that our findings cannot be generalised to women.

In conclusion, a short-term HFD in overweight/obese men induced marked alterations in serum metabolite profiles, including changes in the circulating levels of several lipid and amino acid metabolites. Exercising in the morning or evening induced similar improvements in cardiorespiratory fitness but nocturnal glycaemic control only improved in the evening exercise group. The HFD-induced changes in metabolic profiles were only partly reversed by evening exercise. Our findings, and those of others [8, 9], suggest that performing exercise in the afternoon or early evening may confer the greatest metabolic health benefits. Optimising both the timing of exercise [7, 8] and meals [31, 42] may have additive effects on the circadian clock and circulating metabolite profiles [39] to further improve metabolic health. The identification of metabolomics ‘signatures’ in response to such challenges is of clinical importance to guide the discovery of diagnostic and mechanistic biochemical biomarkers that monitor perturbations in an individual’s metabolic homeostasis. Applying circadian or time of day principles to exercise training may augment the potency of exercise as a therapeutic tool for patient populations.

## Supplementary Information

ESM (PDF 647 kb)

## Data Availability

The datasets generated and analysed during the current study are available from the corresponding author on request.

## Abbreviations

BCAA: Branch-chained amino acid
CGM: Continuous glucose monitor
CON: Control group (no exercise)
DXA: Dual-energy x-ray absorptiometry
EXam: Morning exercise
EXpm: Evening exercise
HFD: High-fat diet
HIT: High-intensity interval training
MET: Metabolic equivalent of task
βOHB: β-Hydroxybutyrate
PCA: Principal component analysis
PPO: Peak power output
UPLC-MS/MS: Ultra-HPLC–tandem MS
$\dot{V}{\mathrm{O}}_{2\mathrm{peak}}$: Peak oxygen uptake

## References

[1] Jensen MD, Ryan DH, Apovian CM (2014). 2013 AHA/ACC/TOS guideline for the management of overweight and obesity in adults: a report of the American College of Cardiology/American Heart Association Task Force on Practice Guidelines and The Obesity Society. Circulation.

[2] Locke A, Schneiderhan J, Zick SM (2018). Diets for health: goals and guidelines. Am Fam Physician.

[3] Garber CE, Blissmer B, Deschenes MR (2011). American College of Sports Medicine position stand. Quantity and quality of exercise for developing and maintaining cardiorespiratory, musculoskeletal, and neuromotor fitness in apparently healthy adults: guidance for prescribing exercise. Med Sci Sports Exerc.

[4] Ezagouri S, Zwighaft Z, Sobel J (2019). Physiological and molecular dissection of daily variance in exercise capacity. Cell Metab.

[5] Wolff G, Esser KA (2012). Scheduled exercise phase shifts the circadian clock in skeletal muscle. Med Sci Sports Exerc.

[6] Sato S, Basse AL, Schonke M (2019). Time of exercise specifies the impact on muscle metabolic pathways and systemic energy homeostasis. Cell Metab.

[7] Erickson ML, Esser KA, Kraus WE, Buford TW, Redman LM (2021). A role for exercise to counter skeletal muscle clock disruption. Exerc Sport Sci Rev.

[8] Savikj M, Gabriel BM, Alm PS (2019). Afternoon exercise is more efficacious than morning exercise at improving blood glucose levels in individuals with type 2 diabetes: a randomised crossover trial. Diabetologia.

[9] Mancilla R, Brouwers B, Schrauwen-Hinderling VB, Hesselink MKC, Hoeks J, Schrauwen P (2021). Exercise training elicits superior metabolic effects when performed in the afternoon compared to morning in metabolically compromised humans. Physiol Rep.

[10] Teo SYM, Kanaley JA, Guelfi KJ, Marston KJ, Fairchild TJ (2020). The effect of exercise timing on glycemic control: a randomized clinical trial. Med Sci Sports Exerc.

[11] Matthews DR, Hosker JP, Rudenski AS, Naylor BA, Treacher DF, Turner RC (1985). Homeostasis model assessment: insulin resistance and beta-cell function from fasting plasma glucose and insulin concentrations in man. Diabetologia.

[12] Evans AM, DeHaven CD, Barrett T, Mitchell M, Milgram E (2009). Integrated, nontargeted ultrahigh performance liquid chromatography/electrospray ionization tandem mass spectrometry platform for the identification and relative quantification of the small-molecule complement of biological systems. Anal Chem.

[13] Dehaven CD, Evans AM, Dai H, Lawton KA (2010). Organization of GC/MS and LC/MS metabolomics data into chemical libraries. J Cheminform.

[14] Twisk J, Bosman L, Hoekstra T, Rijnhart J, Welten M, Heymans M (2018). Different ways to estimate treatment effects in randomised controlled trials. Contemp Clin Trials Commun.

[15] Benjamini Y, Hochberg Y (1995). Controlling the false discovery rate: a practical and powerful approach to multiple testing. J Roy Stat Soc.

[16] Sato S, Parr EB, Devlin BL, Hawley JA, Sassone-Corsi P (2018). Human metabolomics reveal daily variations under nutritional challenges specific to serum and skeletal muscle. Mol Metab.

[17] Kerner J, Hoppel C (2000). Fatty acid import into mitochondria. Biochim Biophys Acta.

[18] Chocian G, Chabowski A, Zendzian-Piotrowska M, Harasim E, Łukaszuk B, Górski J (2010). High fat diet induces ceramide and sphingomyelin formation in rat’s liver nuclei. Mol Cell Biochem.

[19] Siddique MM, Li Y, Chaurasia B, Kaddai VA, Summers SA (2015). Dihydroceramides: from bit players to lead actors. J Biol Chem.

[20] Choi S, Snider AJ (2015). Sphingolipids in high fat diet and obesity-related diseases. Mediat Inflamm.

[21] Chaurasia B, Summers SA (2015). Ceramides - lipotoxic inducers of metabolic disorders. Trends Endocrinol Metab.

[22] Held NM, Wefers J, van Weeghel M (2020). Skeletal muscle in healthy humans exhibits a day-night rhythm in lipid metabolism. Mol Metab.

[23] Loizides-Mangold U, Perrin L, Vandereycken B (2017). Lipidomics reveals diurnal lipid oscillations in human skeletal muscle persisting in cellular myotubes cultured in vitro. Proc Natl Acad Sci U S A.

[24] Burke LM (2021). Ketogenic low-CHO, high-fat diet: the future of elite endurance sport?. J Physiol.

[25] Newman JC, Verdin E (2017). β-Hydroxybutyrate: a signaling metabolite. Annu Rev Nutr.

[26] Koutnik AP, D'Agostino DP, Egan B (2019). Anticatabolic effects of ketone bodies in skeletal muscle. Trends Endocrinol Metab.

[27] Felig P, Marliss E, Cahill GF (1969). Plasma amino acid levels and insulin secretion in obesity. N Engl J Med.

[28] Yang Q, Vijayakumar A, Kahn BB (2018). Metabolites as regulators of insulin sensitivity and metabolism. Nat Rev Mol Cell Biol.

[29] Wang TJ, Larson MG, Vasan RS (2011). Metabolite profiles and the risk of developing diabetes. Nat Med.

[30] Guasch-Ferré M, Hruby A, Toledo E (2016). Metabolomics in prediabetes and diabetes: a systematic review and meta-analysis. Diabetes Care.

[31] Hawley JA, Sassone-Corsi P, Zierath JR (2020). Chrono-nutrition for the prevention and treatment of obesity and type 2 diabetes: from mice to men. Diabetologia.

[32] Green HJ, Helyar R, Ball-Burnett M, Kowalchuk N, Symon S, Farrance B (1992). Metabolic adaptations to training precede changes in muscle mitochondrial capacity. J Appl Physiol (1985).

[33] Burgomaster KA, Howarth KR, Phillips SM (2008). Similar metabolic adaptations during exercise after low volume sprint interval and traditional endurance training in humans. J Physiol.

[34] Burgomaster KA, Hughes SC, Heigenhauser GJ, Bradwell SN, Gibala MJ (2005). Six sessions of sprint interval training increases muscle oxidative potential and cycle endurance capacity in humans. J Appl Physiol (1985).

[35] van Moorsel D, Hansen J, Havekes B (2016). Demonstration of a day-night rhythm in human skeletal muscle oxidative capacity. Mol Metab.

[36] Facer-Childs E, Brandstaetter R (2015). The impact of circadian phenotype and time since awakening on diurnal performance in athletes. Curr Biol.

[37] Van Cauter E, Polonsky KS, Scheen AJ (1997). Roles of circadian rhythmicity and sleep in human glucose regulation. Endocr Rev.

[38] Jarrett RJ, Keen H (1969). Diurnal variation of oral glucose tolerance: a possible pointer to the evolution of diabetes mellitus. BMJ.

[39] Lundell LS, Parr EB, Devlin BL (2020). Time-restricted feeding alters lipid and amino acid metabolite rhythmicity without perturbing clock gene expression. Nat Commun.

[40] Rao MN, Neylan TC, Grunfeld C, Mulligan K, Schambelan M, Schwarz JM (2015). Subchronic sleep restriction causes tissue-specific insulin resistance. J Clin Endocrinol Metab.

[41] Saner NJ, Lee MJC, Kuang J (2021). Exercise mitigates sleep-loss-induced changes in glucose tolerance, mitochondrial function, sarcoplasmic protein synthesis, and diurnal rhythms. Mol Metab.

[42] Parr EB, Heilbronn LK, Hawley JA (2020). A time to eat and a time to exercise. Exerc Sport Sci Rev.

