# Supplementary material for: The effect of morning vs evening exercise training on glycaemic control and serum metabolites in overweight/obese men: a randomised trial
Source: Diabetologia. 2021 May 19;64(9):2061–76. doi: 10.1007/s00125-021-05477-5 (PMC8382617; doi:10.1007/s00125-021-05477-5)
Supplement: Supplementary file 1 — (PDF 647 kb) [file 125_2021_5477_MOESM1_ESM.pdf]

ESM Table 1. Time in bed, according to group, as estimated by ActivPal inclinometer. Values are mean ± SD. *P* values are from paired t-tests.

Group	5-d high-fat diet	Exercise/no exercise	*p* value
**Control**	8.2 ± 1.4	8.4 ± 1.3	0.62
**EXam**	7.7 ± 1.3	7.3 ± 1.0	0.20
**EXpm**	8.3 ± 0.8	8.9 ± 1.2	0.22

ESM Table 2. Fasting and postprandial circulating markers of metabolic health and blood pressure before and after five days of high fat diet. Data are observed means ± SD. *P* values from paired samples t-tests.

	Habitual diet (*n*=24)	High fat diet (*n*=24)	*p* value
**Fasting**			
Glucose (mmol/l)	4.9 ± 0.4	4.8 ± 0.3	0.07
Insulin (pmol/l)	69 ± 44	71 ± 47	0.77
HOMA-IR	2.21 ± 1.5	2.16 ± 1.6	0.96
Cholesterol (mmol/l)	4.8 ± 0.8	4.8 ± 0.8	0.60
Triacylglycerol (mmol/l)	1.54 ± 0.7	1.25 ± 0.6	**0.03**
HDL (mmol/l)	1.04 ± 0.2	1.05 ± 0.1	0.63
LDL (mmol/l)	3.0 ± 0.7	3.2 ± 0.7	**0.049**
Systolic blood pressure (mmHg)	130 ± 9	126 ± 10	0.06
Diastolic blood pressure (mmHg)	84 ± 6	82 ± 5	0.06
**Postprandial**			
Glucose (mmol/l)	5.1 ± 1.0	5.1 ± 0.5	0.92
Insulin (pmol/l)	432 ± 420	328 ± 243	0.15
Cholesterol (mmol/l)	4.8 ± 0.8	5.0 ± 0.8	0.08
Triacylglycerol (mmol/l)	2.4 ± 1.2	2.3 ± 1.0	0.52
HDL (mmol/l)	1.0 ± 0.2	1.1 ± 0.1	0.06
LDL (mmol/l)	2.7 ± 0.8	2.9 ± 0.7	0.18

ESM Table 3. Metabolites significantly changed in morning samples after 5 d high-fat diet.

	**change**	***p* value**	***q value***
(N(1) + N(8))-acetylspermidine	15%	0.0022	0.0090
1-(1-enyl-palmitoyl)-2-arachidonoyl-GPE (P-16:0/20:4)	17%	0.0099	0.0294
1-(1-enyl-palmitoyl)-GPC (P-16:0)	-12%	0.0011	0.0053
1-(1-enyl-stearoyl)-2-arachidonoyl-GPE (P-18:0/20:4)	29%	<0.0001	<0.0001
1.2-dilinoleoyl-GPC (18:2/18:2)	-20%	<0.0001	0.0003
1.2-dilinoleoyl-GPE (18:2/18:2)	-13%	0.0037	0.0137
1.2-dipalmitoyl-GPE (16:0/16:0)	-29%	0.0006	0.0031
1.3-dimethylurate	38%	0.0098	0.0293
1.5-anhydroglucitol (1.5-AG)	-3%	0.0093	0.0280
1.7-dimethylurate	32%	0.0014	0.0065
10-nonadecenoate (19:1n9)	42%	0.0043	0.0152
10-undecenoate (11:1n1)	48%	0.0040	0.0145
12.13-DiHOME	697%	<0.0001	0.0001
1-arachidonoyl-GPE (20:4n6)	-7%	0.0173	0.0463
1-carboxyethylisoleucine	-38%	<0.0001	0.0001
1-carboxyethylleucine	-13%	0.0147	0.0407
1-carboxyethylphenylalanine	-35%	<0.0001	0.0001
1-carboxyethylvaline	-22%	0.0001	0.0006
1-lignoceroyl-GPC (24:0)	-27%	0.0002	0.0015
1-linolenoyl-GPC (18:3)	-45%	<0.0001	0.0003
1-linoleoyl-2-arachidonoyl-GPC (18:2/20:4n6)	-9%	0.0023	0.0095
1-linoleoyl-2-linolenoyl-GPC (18:2/18:3)	-37%	0.0001	0.0006
1-linoleoyl-GPC (18:2)	-11%	0.0010	0.0046
1-linoleoyl-GPE (18:2)	-24%	0.0007	0.0035
1-linoleoyl-GPI (18:2)	-19%	0.0119	0.0344
1-methylhistidine	-7%	0.0078	0.0246
1-methylurate	-17%	0.0044	0.0153
1-methylxanthine	8%	0.0120	0.0344
1-myristoyl-2-arachidonoyl-GPC (14:0/20:4)	-15%	0.0020	0.0084
1-oleoyl-GPC (18:1)	-12%	0.0002	0.0013
1-oleoyl-GPE (18:1)	-21%	0.0017	0.0076
1-palmitoleoyl-2-linolenoyl-GPC (16:1/18:3)	65%	0.0014	0.0064
1-palmitoleoyl-GPC (16:1)	-26%	<0.0001	<0.0001
1-palmitoyl-2-arachidonoyl-GPI (16:0/20:4)	-27%	0.0007	0.0034
1-palmitoyl-2-linoleoyl-GPE (16:0/18:2)	-22%	0.0004	0.0022
1-palmitoyl-2-linoleoyl-GPI (16:0/18:2)	-30%	0.0001	0.0008
1-palmitoyl-2-oleoyl-GPC (16:0/18:1)	-8%	0.0027	0.0103
1-palmitoyl-2-oleoyl-GPE (16:0/18:1)	-23%	0.0001	0.0008
1-palmitoyl-2-palmitoleoyl-GPC (16:0/16:1)	-28%	<0.0001	0.0003
1-palmitoyl-GPC (16:0)	-15%	0.0001	0.0009
1-palmitoyl-GPE (16:0)	-21%	0.0001	0.0008
1-ribosyl-imidazoleacetate	-23%	0.0007	0.0034
1-stearoyl-2-linoleoyl-GPE (18:0/18:2)	-14%	0.0122	0.0346

1-stearoyl-2-linoleoyl-GPI (18:0/18:2)	-18%	0.0003	0.0019
1-stearoyl-GPC (18:0)	-13%	<0.0001	0.0004
1-stearoyl-GPE (18:0)	-20%	<0.0001	0.0002
2.3-dihydroxy-5-methylthio-4-pentenoate (DMTPA)	-7%	<0.0001	0.0003
2.3-dihydroxyisovalerate	-39%	<0.0001	0.0003
21-hydroxypregnenolone disulfate	12%	0.0168	0.0455
2-acetamidophenol sulfate	-35%	0.0001	0.0006
2-aminoadipate	20%	0.0002	0.0011
2-aminobutyrate	75%	<0.0001	<0.0001
2-aminophenol sulfate	-59%	<0.0001	0.0004
2-hydroxy-3-methylvalerate	14%	0.0128	0.0362
2-hydroxybutyrate/2-hydroxyisobutyrate	93%	<0.0001	<0.0001
2-hydroxyglutarate	-20%	<0.0001	<0.0001
2-linoleoylglycerol (18:2)	-31%	0.0085	0.0263
2-methylbutyrylcarnitine (C5)	40%	<0.0001	<0.0001
2-methylcitrate/homocitrate	39%	<0.0001	0.0001
2-oxoarginine	-25%	<0.0001	0.0001
2-palmitoleoyl-GPC (16:1)	-23%	0.0064	0.0209
3-(3-hydroxyphenyl)propionate	31%	0.0063	0.0206
3-(4-hydroxyphenyl)lactate	-9%	0.0040	0.0144
3.7-dimethylurate	361%	0.0106	0.0314
3-acetylphenol sulfate	600%	0.0088	0.0271
3-aminoisobutyrate	25%	0.0007	0.0036
3-carboxy-4-methyl-5-pentyl-2-furanpropionate (3-CMPFP)	50%	<0.0001	<0.0001
3-hydroxy-2-ethylpropionate	63%	<0.0001	<0.0001
3-hydroxyadipate	180%	0.0020	0.0082
beta-hydroxybutyrate (βOHB)	224%	<0.0001	0.0001
3-hydroxybutyroylglycine	125%	<0.0001	0.0002
3-hydroxybutyrylcarnitine (1)	455%	<0.0001	<0.0001
3-hydroxybutyrylcarnitine (2)	73%	<0.0001	<0.0001
3-hydroxydecanoate	99%	<0.0001	<0.0001
3-hydroxyhexanoate	86%	<0.0001	<0.0001
3-hydroxyhippurate	-36%	0.0024	0.0098
3-hydroxyisobutyrate	60%	0.0002	0.0011
3-hydroxylaurate	95%	<0.0001	<0.0001
3-hydroxyoctanoate	81%	<0.0001	<0.0001
3-hydroxypyridine sulfate	-12%	0.0037	0.0136
3-hydroxysebacate	234%	<0.0001	<0.0001
3-methoxycatechol sulfate (2)	-31%	0.0005	0.0029
3-methyl catechol sulfate (1)	17%	0.0178	0.0474
3-methyl-2-oxobutyrate	19%	0.0004	0.0021
3-methyl-2-oxovalerate	17%	0.0016	0.0071
3-methylglutaconate	15%	0.0022	0.0092
3-methylhistidine	-36%	0.0004	0.0023
3-methylxanthine	271%	0.0174	0.0463

3-ureidopropionate	18%	0.0024	0.0096
4-acetylphenol sulfate	-44%	<0.0001	0.0004
4-allylphenol sulfate	-51%	0.0026	0.0103
4-ethylphenylsulfate	-27%	0.0057	0.0190
4-guanidinobutanoate	-33%	0.0001	0.0005
4-hydroxyhippurate	-27%	0.0018	0.0076
4-hydroxyphenylpyruvate	-17%	<0.0001	0.0001
4-methoxyphenol sulfate	-73%	0.0001	0.0008
4-methyl-2-oxopentanoate	18%	0.0014	0.0065
4-methylhexanoylglutamine	320%	<0.0001	<0.0001
4-vinylphenol sulfate	-66%	0.0050	0.0171
5-acetylamino-6-amino-3-methyluracil	13%	0.0026	0.0102
5alpha-androstan-3beta.17beta-diol disulfate	-11%	0.0004	0.0023
5alpha-androstan-3beta.17beta-diol monosulfate (2)	-10%	0.0032	0.0122
5alpha-pregnan-3beta.20alpha-diol disulfate	30%	0.0030	0.0114
5alpha-pregnan-3beta.20alpha-diol monosulfate (2)	42%	0.0120	0.0344
5-dodecenoate (12:1n7)	72%	0.0001	0.0004
5-dodecenoylcarnitine (C12:1)	101%	0.0001	0.0007
7-methylurate	188%	0.0120	0.0344
7-methylxanthine	245%	0.0086	0.0264
acetoacetate	340%	<0.0001	0.0004
acetylcarnitine (C2)	54%	<0.0001	<0.0001
acisoga	41%	0.0008	0.0039
adipoylcarnitine (C6-DC)	80%	<0.0001	0.0001
alanine	-10%	0.0173	0.0463
alpha-hydroxyisocaproate	19%	0.0030	0.0115
alpha-hydroxyisovalerate	16%	0.0043	0.0153
alpha-ketobutyrate	177%	0.0002	0.0012
alpha-ketoglutaramate	-9%	0.0099	0.0294
androstenediol (3beta.17beta) disulfate (1)	-14%	<0.0001	<0.0001
androstenediol (3beta.17beta) monosulfate (2)	-11%	<0.0001	0.0004
arabitol/xylitol	-21%	0.0001	0.0006
arabonate/xylonate	-13%	0.0050	0.0170
argininate	-15%	0.0016	0.0071
arginine	-4%	0.0071	0.0226
aspartate	15%	0.0069	0.0221
behenoyl dihydrosphingomyelin (d18:0/22:0)	31%	<0.0001	0.0002
benzoylcarnitine	84%	<0.0001	0.0001
beta-hydroxyisovalerate	42%	<0.0001	<0.0001
betaine	8%	0.0007	0.0035
caffeine	36%	0.0015	0.0069
caproate (6:0)	20%	0.0025	0.0100
carboxyethyl-GABA	-18%	0.0078	0.0245
carotene diol (1)	27%	0.0046	0.0159
ceramide (d18:1/17:0. d17:1/18:0)	72%	<0.0001	<0.0001

ceramide (d18:1/20:0. d16:1/22:0. d20:1/18:0)	-7%	0.0159	0.0433
chenodeoxycholate	-22%	0.0121	0.0346
cis-4-decenoate (10:1n6)	38%	0.0008	0.0038
cis-4-decenoylcarnitine (C10:1)	43%	0.0182	0.0481
citrate	28%	0.0003	0.0018
creatine	10%	0.0095	0.0285
decanoylcarnitine (C10)	121%	<0.0001	<0.0001
diacylglycerol (14:0/18:1. 16:0/16:1) [1]	-33%	0.0045	0.0157
diacylglycerol (14:0/18:1. 16:0/16:1) [2]	-21%	0.0145	0.0405
diacylglycerol (16:1/18:2 [2]. 16:0/18:3 [1])	-33%	0.0007	0.0034
dihomo-linolenoylcarnitine (C20:3n3 or 6)	-10%	0.0071	0.0226
dihydroorotate	8%	0.0079	0.0248
dimethylglycine	13%	0.0001	0.0009
docosahexaenoylcarnitine (C22:6)	77%	<0.0001	<0.0001
dodecadienoate (12:2)	46%	0.0029	0.0112
dodecenedioate (C12:1-DC)	108%	<0.0001	<0.0001
dopamine 3-O-sulfate	-21%	0.0011	0.0051
dopamine 4-sulfate	-15%	0.0052	0.0174
eicosanedioate (C20-DC)	-36%	0.0003	0.0018
eicosapentaenoylcholine	5%	0.0019	0.0081
erythritol	944%	0.0001	0.0006
Fibrinopeptide A (7-16)	39%	0.0130	0.0366
galactonate	47%	0.0073	0.0232
gamma-CEHC	-29%	0.0001	0.0004
gamma-glutamyl-2-aminobutyrate	109%	<0.0001	<0.0001
gamma-glutamylglycine	-10%	0.0067	0.0216
gamma-glutamylhistidine	-9%	0.0013	0.0059
gamma-glutamylleucine	11%	0.0148	0.0407
gamma-glutamylthreonine	-11%	0.0158	0.0430
gamma-glutamyltryptophan	-9%	0.0016	0.0072
gamma-glutamyltyrosine	-11%	0.0042	0.0149
gamma-glutamylvaline	18%	0.0114	0.0332
gamma-tocopherol/beta-tocopherol	-48%	<0.0001	<0.0001
glucose	-2%	0.0189	0.0493
glutamine	-4%	0.0134	0.0374
glycerol	45%	0.0003	0.0018
glycerol 3-phosphate	-7%	0.0038	0.0141
glycerophosphorylcholine (GPC)	-14%	0.0007	0.0035
glycine	-7%	0.0066	0.0214
glycine conjugate of C10H14O2 (1)	-64%	<0.0001	0.0004
glyco-beta-muricholate	207%	0.0039	0.0141
glycosyl ceramide (d18:1/23:1. d17:1/24:1)	31%	0.0001	0.0004
glycosyl ceramide (d18:2/24:1. d18:1/24:2)	10%	0.0049	0.0168
glycosyl-N-nervonoyl-sphingosine (d18:1/24:1)	17%	0.0189	0.0493
glycosyl-N-palmitoyl-sphingosine (d18:1/16:0)	21%	<0.0001	0.0001

glycosyl-N-stearoyl-sphingosine (d18:1/18:0)	11%	0.0020	0.0082
glycosyl-N-tricosanoyl-sphingadienine (d18:2/23:0)	30%	<0.0001	<0.0001
guaiacol sulfate	-14%	0.0116	0.0339
guanidinoacetate	-11%	0.0002	0.0011
guanidinosuccinate	34%	<0.0001	<0.0001
heptenedioate (C7:1-DC)	206%	<0.0001	0.0003
hexadecanedioate (C16-DC)	30%	0.0004	0.0023
hexanoylcarnitine (C6)	75%	<0.0001	<0.0001
hexanoylglutamine	154%	<0.0001	0.0001
hexanoylglycine	125%	0.0146	0.0405
homoarginine	16%	0.0005	0.0025
hydroxypalmitoyl sphingomyelin (d18:1/16:0(OH))	9%	0.0091	0.0277
indole-3-carboxylate	36%	0.0017	0.0074
indoleacetate	20%	0.0045	0.0158
indoleacetylcarnitine	62%	0.0001	0.0009
isoleucine	7%	0.0053	0.0175
isovalerylcarnitine (C5)	28%	0.0006	0.0031
lactate	-13%	0.0019	0.0081
laurylcarnitine (C12)	118%	<0.0001	<0.0001
leucine	7%	0.0092	0.0278
linolenoylcarnitine (C18:3)	-22%	0.0009	0.0042
linoleoyl-arachidonoyl-glycerol (18:2/20:4) [2]	-24%	0.0168	0.0455
linoleoyl-linolenoyl-glycerol (18:2/18:3) [1]	-52%	0.0007	0.0035
linoleoyl-linoleoyl-glycerol (18:2/18:2) [1]	-13%	0.0062	0.0205
malate	10%	0.0131	0.0370
mannitol/sorbitol	-26%	0.0182	0.0481
mannose	26%	<0.0001	<0.0001
margaroylcarnitine (C17)	65%	<0.0001	<0.0001
methionine	-7%	0.0052	0.0174
myristoleoylcarnitine (C14:1)	120%	<0.0001	<0.0001
myristoyl dihydrosphingomyelin (d18:0/14:0)	54%	<0.0001	<0.0001
myristoylcarnitine (C14)	68%	<0.0001	<0.0001
myristoylcholine	-21%	0.0002	0.0011
N1-methylinosine	10%	0.0085	0.0263
N6.N6-dimethyllysine	-5%	0.0172	0.0462
N-acetyl-3-methylhistidine	-35%	0.0001	0.0008
N-acetylglycine	70%	<0.0001	0.0001
N-acetylmethionine	-12%	<0.0001	0.0001
N-acetylphenylalanine	-10%	0.0007	0.0034
N-acetylproline	-16%	0.0042	0.0149
N-acetylputrescine	-7%	0.0111	0.0327
N-acetyltaurine	-7%	0.0154	0.0422
N-acetyltryptophan	-22%	<0.0001	0.0002
N-acetyltyrosine	-15%	0.0007	0.0036
N-behenoyl-sphingadienine (d18:2/22:0)	-11%	0.0019	0.0080

nonanoylcarnitine (C9)	125%	<0.0001	<0.0001
N-stearoyl-sphingadienine (d18:2/18:0)	441%	0.0004	0.0021
N-stearoyl-sphinganine (d18:0/18:0)	135%	<0.0001	0.0003
N-stearoyl-sphingosine (d18:1/18:0)	31%	0.0001	0.0004
octadecadienedioate (C18:2-DC)	127%	0.0007	0.0036
octadecanedioate (C18-DC)	99%	<0.0001	<0.0001
octadecanedioylcarnitine (C18-DC)	81%	<0.0001	<0.0001
octadecenedioylcarnitine (C18:1-DC)	55%	<0.0001	0.0003
octanoylcarnitine (C8)	110%	<0.0001	0.0002
oleate/vaccenate (18:1)	34%	0.0036	0.0136
oleoylcarnitine (C18:1)	22%	0.0004	0.0021
oleoylcholine	-11%	0.0041	0.0147
orotidine	-19%	0.0048	0.0166
palmitoleate (16:1n7)	19%	0.0091	0.0277
palmitoleoylcarnitine (C16:1)	48%	<0.0001	<0.0001
palmitoleoyl-linoleoyl-glycerol (16:1/18:2) [1]	-29%	0.0008	0.0039
palmitoloelycholine	-23%	0.0001	0.0008
palmitoyl dihydrosphingomyelin (d18:0/16:0)	25%	<0.0001	<0.0001
palmitoyl sphingomyelin (d18:1/16:0)	9%	<0.0001	0.0002
palmitoyl-arachidonoyl-glycerol (16:0/20:4) [1]	-11%	0.0133	0.0374
palmitoylcarnitine (C16)	16%	0.0016	0.0071
pantothenate	11%	0.0008	0.0039
paraxanthine	-15%	0.0002	0.0010
phenylacetylcarnitine	73%	0.0030	0.0114
phenyllactate (PLA)	-7%	0.0019	0.0080
phytanate	194%	<0.0001	<0.0001
picolinate	24%	0.0003	0.0017
pipecolate	30%	0.0110	0.0324
pregnanediol-3-glucuronide	53%	0.0025	0.0100
pristanate	634%	<0.0001	0.0003
pro-hydroxy-pro	15%	0.0051	0.0172
proline	-14%	<0.0001	0.0001
propionylcarnitine (C3)	20%	0.0023	0.0095
pyrraline	-50%	<0.0001	<0.0001
pyruvate	-23%	0.0002	0.0013
ribitol	-13%	0.0003	0.0019
ribonate	-10%	0.0032	0.0119
serine	11%	0.0009	0.0043
S-methylcysteine	113%	<0.0001	<0.0001
S-methylcysteine sulfoxide	228%	<0.0001	<0.0001
S-methylmethionine	384%	0.0028	0.0107
sphingomyelin (d17:1/14:0. d16:1/15:0)	17%	0.0037	0.0136
sphingomyelin (d17:1/16:0. d18:1/15:0. d16:1/17:0)	16%	<0.0001	<0.0001
sphingomyelin (d17:2/16:0. d18:2/15:0)	13%	0.0014	0.0065
sphingomyelin (d18:0/18:0. d19:0/17:0)	98%	<0.0001	<0.0001

sphingomyelin (d18:0/20:0. d16:0/22:0)	51%	<0.0001	<0.0001
sphingomyelin (d18:1/14:0. d16:1/16:0)	15%	<0.0001	0.0002
sphingomyelin (d18:1/17:0. d17:1/18:0. d19:1/16:0)	32%	<0.0001	<0.0001
sphingomyelin (d18:1/18:1. d18:2/18:0)	26%	<0.0001	<0.0001
sphingomyelin (d18:1/19:0. d19:1/18:0)	22%	0.0007	0.0035
sphingomyelin (d18:1/20:1. d18:2/20:0)	10%	0.0051	0.0172
sphingomyelin (d18:1/20:2. d18:2/20:1. d16:1/22:2)	30%	0.0003	0.0018
sphingomyelin (d18:1/21:0. d17:1/22:0. d16:1/23:0)	14%	<0.0001	0.0003
sphingomyelin (d18:1/22:2. d18:2/22:1. d16:1/24:2)	26%	<0.0001	0.0002
sphingomyelin (d18:1/25:0. d19:0/24:1. d20:1/23:0. d19:1/24:0)	36%	<0.0001	<0.0001
sphingomyelin (d18:2/16:0. d18:1/16:1)	7%	0.0062	0.0204
sphingomyelin (d18:2/18:1)	51%	<0.0001	<0.0001
sphingomyelin (d18:2/21:0. d16:2/23:0)	30%	<0.0001	<0.0001
sphingomyelin (d18:2/23:0. d18:1/23:1. d17:1/24:1)	19%	<0.0001	<0.0001
sphingomyelin (d18:2/23:1)	30%	<0.0001	<0.0001
sphingomyelin (d18:2/24:2)	13%	0.0026	0.0102
stearoyl sphingomyelin (d18:1/18:0)	33%	<0.0001	<0.0001
stearoylcarnitine (C18)	50%	<0.0001	<0.0001
suberoylcarnitine (C8-DC)	220%	0.0021	0.0085
succinylcarnitine (C4-DC)	-16%	0.0001	0.0004
tetradecadienoate (14:2)	58%	0.0001	0.0006
theobromine	484%	0.0150	0.0413
theophylline	32%	0.0002	0.0010
threonine	-13%	<0.0001	0.0003
tiglylcarnitine (C5:1-DC)	54%	<0.0001	<0.0001
tricosanoyl sphingomyelin (d18:1/23:0)	19%	<0.0001	<0.0001
trimethylamine N-oxide	71%	0.0001	0.0004
tryptophan	-5%	0.0065	0.0213
tyrosine	-11%	<0.0001	0.0002
urea	19%	0.0003	0.0018
uridine	23%	<0.0001	0.0001
ursodeoxycholate	-9%	0.0067	0.0216
valine	7%	0.0082	0.0257
vanillactate	-8%	0.0113	0.0332
xanthine	23%	0.0185	0.0486

ESM Table 4. Metabolites significantly changed in evening samples after 5 d high-fat diet.

	**change**	***p*** **value**	***q value***
(N(1) + N(8))-acetylspermidine	-8%	0.0081	0.0206
1-(1-enyl-oleoyl)-GPE (P-18:1)	26%	0.0142	0.0339
1-(1-enyl-palmitoyl)-2-arachidonoyl-GPE (P-16:0/20:4)	34%	0.0002	0.0011
1-(1-enyl-palmitoyl)-2-oleoyl-GPE (P-16:0/18:1)	24%	0.0190	0.0430
1-(1-enyl-palmitoyl)-2-palmitoyl-GPC (P-16:0/16:0)	6%	0.0110	0.0271
1-(1-enyl-palmitoyl)-GPC (P-16:0)	-16%	0.0001	0.0003
1-(1-enyl-stearoyl)-2-arachidonoyl-GPE (P-18:0/20:4)	50%	<0.0001	<0.0001
1-(1-enyl-stearoyl)-2-linoleoyl-GPE (P-18:0/18:2)	30%	0.0002	0.0008
1-(1-enyl-stearoyl)-2-oleoyl-GPE (P-18:0/18:1)	33%	0.0001	0.0006
1.2-dilinoleoyl-GPC (18:2/18:2)	-11%	0.0049	0.0139
1.2-dipalmitoyl-GPC (16:0/16:0)	11%	0.0002	0.0007
1.5-anhydroglucitol (1.5-AG)	-3%	0.0044	0.0127
10-nonadecenoate (19:1n9)	49%	<0.0001	0.0002
10-undecenoate (11:1n1)	51%	0.0012	0.0042
12.13-DiHOME	831%	<0.0001	<0.0001
13-HODE + 9-HODE	149%	<0.0001	0.0001
1-arachidonoyl-GPC (20:4n6)	8%	0.0002	0.0011
1-arachidonoyl-GPE (20:4n6)	17%	0.0003	0.0012
1-arachidonylglycerol (20:4)	84%	0.0009	0.0031
1-docosahexaenoylglycerol (22:6)	133%	0.0002	0.0007
1-lignoceroyl-GPC (24:0)	-30%	<0.0001	<0.0001
1-linolenoyl-GPC (18:3)	-22%	0.0010	0.0036
1-linoleoyl-2-linolenoyl-GPC (18:2/18:3)	-33%	0.0002	0.0011
1-methylhistidine	-14%	0.0009	0.0033
1-methylnicotinamide	74%	0.0047	0.0136
1-myristoyl-2-arachidonoyl-GPC (14:0/20:4)	29%	0.0100	0.0249
1-myristoyl-2-palmitoyl-GPC (14:0/16:0)	54%	0.0036	0.0107
1-palmitoleoyl-2-linolenoyl-GPC (16:1/18:3)	-17%	0.0017	0.0055
1-palmitoleoyl-GPC (16:1)	-18%	0.0001	0.0005
1-palmitoyl-2-arachidonoyl-GPC (16:0/20:4n6)	9%	0.0062	0.0167
1-palmitoyl-2-arachidonoyl-GPI (16:0/20:4)	-12%	0.0183	0.0419
1-palmitoyl-2-dihomo-linolenoyl-GPC (16:0/20:3n3 or 6)	-5%	0.0051	0.0143
1-palmitoyl-2-docosahexaenoyl-GPC (16:0/22:6)	9%	0.0021	0.0067
1-palmitoyl-2-linoleoyl-GPI (16:0/18:2)	-23%	<0.0001	0.0003
1-palmitoyl-2-palmitoleoyl-GPC (16:0/16:1)	-15%	0.0015	0.0052
1-palmitoyl-GPC (16:0)	-7%	0.0042	0.0123
1-palmitoyl-GPE (16:0)	-15%	0.0001	0.0007
1-stearoyl-2-arachidonoyl-GPC (18:0/20:4)	10%	0.0017	0.0056
1-stearoyl-2-arachidonoyl-GPE (18:0/20:4)	19%	0.0191	0.0430
1-stearoyl-2-linoleoyl-GPC (18:0/18:2)	7%	0.0139	0.0333
1-stearoyl-2-oleoyl-GPI (18:0/18:1)	25%	0.0132	0.0317
1-stearoyl-GPC (18:0)	-4%	0.0213	0.0469

1-stearoyl-GPE (18:0)	-12%	0.0003	0.0014
2.3-dihydroxy-5-methylthio-4-pentenoate (DMTPA)	12%	0.0171	0.0400
2.3-dihydroxyisovalerate	-84%	0.0001	0.0005
2-aminoadipate	50%	0.0003	0.0012
2-aminobutyrate	104%	<0.0001	<0.0001
2-aminophenol sulfate	-55%	0.0035	0.0105
2-hydroxy-3-methylvalerate	50%	<0.0001	0.0002
2-hydroxybutyrate/2-hydroxyisobutyrate	135%	<0.0001	<0.0001
2-hydroxyglutarate	-18%	<0.0001	<0.0001
2-hydroxyhippurate (salicylurate)	-51%	0.0050	0.0142
2-hydroxynervonate	31%	0.0004	0.0017
2-keto-3-deoxy-gluconate	30%	0.0045	0.0130
2-linoleoylglycerol (18:2)	-37%	0.0011	0.0038
2-methylbutyrylcarnitine (C5)	71%	<0.0001	<0.0001
2-methylcitrate/homocitrate	30%	<0.0001	0.0001
2-oxoarginine	28%	0.0058	0.0160
3-(3-hydroxyphenyl)propionate	-53%	0.0063	0.0170
3-(4-hydroxyphenyl)lactate	28%	<0.0001	<0.0001
3.4-methyleneheptanoate	3%	0.0012	0.0042
3-acetylphenol sulfate	-38%	0.0053	0.0146
3-carboxy-4-methyl-5-pentyl-2-furanpropionate (3-CMPFP)	58%	<0.0001	<0.0001
3-ethylphenylsulfate	1062%	<0.0001	<0.0001
3-formylindole	-7%	0.0179	0.0411
3-hydroxy-2-ethylpropionate	118%	<0.0001	<0.0001
3-hydroxy-3-methylglutarate	-19%	0.0094	0.0237
3-hydroxyadipate	202%	0.0001	0.0006
beta-hydroxybutyrate (βOHB)	522%	<0.0001	<0.0001
3-hydroxybutyroylglycine	366%	<0.0001	<0.0001
3-hydroxybutyrylcarnitine (1)	569%	<0.0001	<0.0001
3-hydroxybutyrylcarnitine (2)	107%	<0.0001	<0.0001
3-hydroxydecanoate	134%	<0.0001	<0.0001
3-hydroxyhexanoate	148%	<0.0001	<0.0001
3-hydroxyhippurate	-54%	0.0024	0.0077
3-hydroxyisobutyrate	120%	<0.0001	<0.0001
3-hydroxylaurate	142%	<0.0001	<0.0001
3-hydroxyoctanoate	97%	<0.0001	<0.0001
3-hydroxyoleate	340%	<0.0001	<0.0001
3-hydroxypyridine sulfate	-33%	0.0114	0.0279
3-hydroxysebacate	470%	<0.0001	<0.0001
3-methoxycatechol sulfate (2)	-53%	0.0001	0.0004
3-methoxytyramine sulfate	-31%	0.0004	0.0018
3-methoxytyrosine	-12%	0.0044	0.0127
3-methyl catechol sulfate (1)	4%	0.0162	0.0380
3-methyl-2-oxobutyrate	35%	<0.0001	<0.0001
3-methyl-2-oxovalerate	24%	0.0001	0.0004

3-methylglutaconate	66%	<0.0001	<0.0001
3-ureidopropionate	-8%	0.0117	0.0285
4-acetylphenol sulfate	-65%	<0.0001	<0.0001
4-allylphenol sulfate	-54%	0.0070	0.0183
4-cholesten-3-one	-15%	0.0042	0.0123
4-guanidinobutanoate	-15%	0.0176	0.0406
4-hydroxyhippurate	-42%	<0.0001	0.0002
4-methoxyphenol sulfate	-80%	0.0007	0.0025
4-methyl-2-oxopentanoate	25%	<0.0001	0.0001
4-methylguaiacol sulfate	40%	0.0089	0.0225
4-methylhexanoylglutamine	459%	<0.0001	<0.0001
4-vinylphenol sulfate	-71%	0.0001	0.0007
5-(galactosylhydroxy)-L-lysine	-10%	0.0070	0.0183
5.6-dihydrouridine	-8%	0.0002	0.0007
5-acetylamino-6-amino-3-methyluracil	-9%	0.0190	0.0430
5alpha-androstan-3alpha.17beta-diol monosulfate (2)	17%	0.0172	0.0401
5alpha-androstan-3beta.17beta-diol disulfate	-13%	0.0011	0.0038
5alpha-pregnan-3beta.20alpha-diol monosulfate (2)	65%	0.0068	0.0182
5-dodecenoate (12:1n7)	574%	<0.0001	<0.0001
5-dodecenoylcarnitine (C12:1)	109%	0.0078	0.0201
5-hydroxyhexanoate	100%	0.0014	0.0048
6-bromotryptophan	-8%	0.0080	0.0205
6-oxopiperidine-2-carboxylate	29%	0.0203	0.0453
9-hydroxystearate	136%	<0.0001	0.0001
acetoacetate	376%	<0.0001	0.0001
acetylcarnitine (C2)	59%	<0.0001	<0.0001
adenosine	103%	0.0052	0.0144
adipate (C6-DC)	416%	<0.0001	0.0002
adipoylcarnitine (C6-DC)	187%	<0.0001	<0.0001
allantoin	-20%	0.0038	0.0114
alliin	-60%	0.0001	0.0003
alpha-hydroxycaproate	165%	0.0053	0.0146
alpha-hydroxyisocaproate	36%	0.0004	0.0017
alpha-hydroxyisovalerate	31%	0.0006	0.0024
alpha-ketobutyrate	262%	<0.0001	0.0002
androstenediol (3beta.17beta) disulfate (1)	-14%	<0.0001	0.0001
androstenediol (3beta.17beta) monosulfate (2)	-14%	<0.0001	0.0001
arabinose	102%	0.0005	0.0020
arabitol/xylitol	-27%	<0.0001	<0.0001
arabonate/xylonate	-26%	0.0006	0.0024
arachidonoylcarnitine (C20:4)	-9%	0.0059	0.0160
argininate	39%	<0.0001	0.0002
behenoyl dihydrosphingomyelin (d18:0/22:0)	46%	<0.0001	<0.0001
benzoylcarnitine	60%	0.0019	0.0060
beta-alanine	79%	0.0004	0.0018

beta-citrylglutamate	22%	0.0004	0.0017
beta-hydroxyisovalerate	83%	<0.0001	<0.0001
caprate (10:0)	636%	<0.0001	<0.0001
caproate (6:0)	56%	0.0002	0.0007
caprylate (8:0)	719%	<0.0001	0.0001
carboxyethyl-GABA	-12%	0.0053	0.0146
carotene diol (1)	41%	<0.0001	0.0001
carotene diol (2)	35%	0.0031	0.0095
carotene diol (3)	33%	0.0068	0.0182
ceramide (d16:1/24:1. d18:1/22:1)	19%	0.0210	0.0466
ceramide (d18:1/14:0. d16:1/16:0)	32%	<0.0001	<0.0001
ceramide (d18:1/17:0. d17:1/18:0)	99%	<0.0001	<0.0001
chenodeoxycholate	185%	0.0008	0.0030
cholate	994%	0.0017	0.0055
choline phosphate	37%	0.0001	0.0005
cis-4-decenoate (10:1n6)	103%	0.0069	0.0183
citrulline	-10%	0.0017	0.0055
cystathionine	72%	0.0019	0.0061
decanoylcarnitine (C10)	186%	<0.0001	<0.0001
diacylglycerol (16:1/18:2 [2]. 16:0/18:3 [1])	-26%	0.0003	0.0012
dihomo-linolenoylcarnitine (C20:3n3 or 6)	-15%	<0.0001	0.0003
dihomo-linolenoyl-choline	-13%	0.0044	0.0127
dihomo-linoleoylcarnitine (C20:2)	-10%	0.0034	0.0103
dimethyl sulfone	-12%	0.0074	0.0194
dimethylglycine	36%	<0.0001	<0.0001
docosahexaenoate (DHA; 22:6n3)	43%	0.0004	0.0018
docosahexaenoylcarnitine (C22:6)	32%	0.0078	0.0201
docosapentaenoate (n6 DPA; 22:5n6)	50%	<0.0001	0.0002
dodecadienoate (12:2)	125%	<0.0001	0.0001
dodecanedioate (C12-DC)	1384%	<0.0001	0.0001
dodecenedioate (C12:1-DC)	210%	<0.0001	0.0001
eicosanedioate (C20-DC)	-33%	0.0001	0.0006
eicosapentaenoylcholine	-11%	0.0027	0.0084
erythritol	1943%	0.0009	0.0032
ethylmalonate	28%	0.0038	0.0114
eugenol sulfate	-70%	0.0051	0.0144
Fibrinopeptide A. des-ala(1)	10%	0.0029	0.0089
Fibrinopeptide B (1-13)	9%	0.0077	0.0200
formiminoglutamate	93%	0.0003	0.0013
fructose	-28%	0.0077	0.0200
gamma-CEHC	-37%	<0.0001	0.0001
gamma-glutamyl-2-aminobutyrate	153%	<0.0001	<0.0001
gamma-glutamylglutamate	23%	0.0046	0.0131
gamma-glutamylglycine	-17%	<0.0001	0.0001
gamma-glutamylhistidine	-7%	0.0112	0.0276

gamma-glutamylisoleucine	35%	<0.0001	<0.0001
gamma-glutamylleucine	39%	<0.0001	<0.0001
gamma-glutamyltryptophan	19%	0.0026	0.0081
gamma-glutamyltyrosine	25%	0.0099	0.0247
gamma-glutamylvaline	55%	<0.0001	<0.0001
gamma-tocopherol/beta-tocopherol	-45%	<0.0001	<0.0001
gentisate	-50%	<0.0001	<0.0001
gluconate	-22%	0.0076	0.0198
glutarylcarnitine (C5-DC)	8%	0.0012	0.0041
glycerol	98%	<0.0001	<0.0001
glycerophosphoinositol	-21%	0.0048	0.0137
glycerophosphorylcholine (GPC)	-11%	0.0004	0.0016
glycine	-13%	0.0001	0.0003
glycine conjugate of C10H14O2 (1)	-76%	<0.0001	<0.0001
glyco-beta-muricholate	124%	<0.0001	0.0001
glycochenodeoxycholate 3-sulfate	77%	0.0001	0.0003
glycodeoxycholate 3-sulfate	75%	0.0006	0.0022
glycosyl ceramide (d18:1/23:1. d17:1/24:1)	35%	<0.0001	<0.0001
glycosyl ceramide (d18:2/24:1. d18:1/24:2)	14%	0.0001	0.0003
glycosyl-N-palmitoyl-sphingosine (d18:1/16:0)	16%	0.0002	0.0008
glycosyl-N-stearoyl-sphingosine (d18:1/18:0)	18%	<0.0001	0.0002
glycosyl-N-tricosanoyl-sphingadienine (d18:2/23:0)	42%	<0.0001	<0.0001
guaiacol sulfate	-38%	0.0026	0.0081
guanidinoacetate	-20%	0.0006	0.0022
guanidinosuccinate	31%	<0.0001	<0.0001
heptenedioate (C7:1-DC)	118%	0.0211	0.0467
hexadecanedioate (C16-DC)	83%	0.0001	0.0003
hexanoylcarnitine (C6)	118%	<0.0001	<0.0001
hexanoylglutamine	410%	<0.0001	<0.0001
hexanoylglycine	332%	<0.0001	<0.0001
hippurate	-23%	0.0071	0.0188
hydantoin-5-propionate	138%	<0.0001	0.0002
hydroquinone sulfate	-30%	0.0117	0.0285
hydroxyasparagine	-9%	<0.0001	0.0001
hyocholate	245%	0.0003	0.0013
indoleacetylcarnitine	39%	<0.0001	0.0002
indolepropionate	-37%	0.0014	0.0047
isobutyrylcarnitine (C4)	76%	<0.0001	0.0001
isocitrate	-20%	0.0002	0.0010
isoleucine	22%	<0.0001	0.0001
isovalerylcarnitine (C5)	55%	0.0002	0.0008
kynurenate	16%	0.0005	0.0020
lactate	-20%	0.0001	0.0005
lactosyl-N-behenoyl-sphingosine (d18:1/22:0)	13%	0.0006	0.0021
lactosyl-N-nervonoyl-sphingosine (d18:1/24:1)	9%	0.0136	0.0325

lanthionine	-24%	0.0093	0.0235
laurate (12:0)	678%	<0.0001	<0.0001
laurylcarnitine (C12)	248%	<0.0001	<0.0001
leucine	24%	<0.0001	0.0001
lignoceroyl sphingomyelin (d18:1/24:0)	13%	0.0003	0.0012
linoleate (18:2n6)	62%	<0.0001	0.0001
linolenoylcarnitine (C18:3)	-24%	0.0003	0.0012
linoleoyl-arachidonoyl-glycerol (18:2/20:4) [1]	-5%	0.0162	0.0381
linoleoyl-arachidonoyl-glycerol (18:2/20:4) [2]	-5%	0.0177	0.0407
linoleoylcarnitine (C18:2)	-10%	0.0040	0.0118
linoleoyl-linolenoyl-glycerol (18:2/18:3) [1]	-48%	0.0012	0.0041
linoleoyl-linoleoyl-glycerol (18:2/18:2) [1]	-26%	0.0018	0.0059
mannitol/sorbitol	-41%	0.0053	0.0146
mannose	67%	<0.0001	<0.0001
margaroylcarnitine (C17)	66%	<0.0001	<0.0001
myristoleoylcarnitine (C14:1)	93%	<0.0001	0.0001
myristoyl dihydrosphingomyelin (d18:0/14:0)	90%	<0.0001	<0.0001
myristoylcarnitine (C14)	135%	<0.0001	<0.0001
N1-methyladenosine	-8%	0.0001	0.0003
N6.N6.N6-trimethyllysine	-25%	0.0017	0.0055
N6.N6-dimethyllysine	-17%	0.0001	0.0003
N6-carbamoylthreonyladenosine	-9%	0.0121	0.0293
N6-carboxymethyllysine	1613%	0.0004	0.0015
N-acetyl-1-methylhistidine	-55%	<0.0001	<0.0001
N-acetyl-2-aminooctanoate	-36%	0.0015	0.0051
N-acetyl-3-methylhistidine	-63%	<0.0001	0.0002
N-acetylarginine	-13%	0.0217	0.0475
N-acetylasparagine	-16%	0.0028	0.0085
N-acetylcarnosine	-6%	0.0161	0.0380
N-acetylglutamate	42%	<0.0001	<0.0001
N-acetylglycine	60%	<0.0001	<0.0001
N-acetylhistidine	10%	0.0010	0.0035
N-acetyl-isoputreanine	-18%	<0.0001	<0.0001
N-acetylneuraminate	11%	0.0005	0.0020
N-acetylphenylalanine	-28%	<0.0001	0.0001
N-acetylproline	-34%	<0.0001	0.0001
N-acetylputrescine	-11%	0.0032	0.0097
N-acetyltaurine	-14%	0.0080	0.0204
N-acetylthreonine	-6%	0.0212	0.0468
N-acetyltryptophan	-41%	<0.0001	<0.0001
N-acetyltyrosine	-20%	0.0024	0.0077
N-behenoyl-sphingadienine (d18:2/22:0)	-12%	0.0013	0.0044
N-carbamoylalanine	-19%	0.0061	0.0165
N-delta-acetylornithine	-20%	0.0051	0.0144
nervonoylcarnitine (C24:1)	27%	0.0209	0.0466

N-oleoyltaurine	128%	0.0089	0.0225
nonanoylcarnitine (C9)	166%	<0.0001	<0.0001
N-palmitoylglycine	11%	0.0203	0.0453
N-palmitoyl-heptadecasphingosine (d17:1/16:0)	31%	<0.0001	0.0001
N-palmitoyl-sphingadienine (d18:2/16:0)	14%	0.0110	0.0271
N-palmitoyl-sphinganine (d18:0/16:0)	81%	<0.0001	<0.0001
N-palmitoyl-sphingosine (d18:1/16:0)	26%	<0.0001	<0.0001
N-stearoyl-sphingadienine (d18:2/18:0)	542%	0.0001	0.0007
N-stearoyl-sphinganine (d18:0/18:0)	243%	<0.0001	<0.0001
N-stearoyl-sphingosine (d18:1/18:0)	49%	<0.0001	<0.0001
o-cresol sulfate	-16%	0.0025	0.0078
octadecanedioate (C18-DC)	128%	<0.0001	0.0002
octadecanedioylcarnitine (C18-DC)	90%	<0.0001	<0.0001
octadecenedioylcarnitine (C18:1-DC)	52%	0.0005	0.0018
octanoylcarnitine (C8)	143%	<0.0001	<0.0001
oleate/vaccenate (18:1)	66%	<0.0001	0.0001
oleoyl-arachidonoyl-glycerol (18:1/20:4) [1]	-8%	0.0216	0.0475
oleoyl-linoleoyl-glycerol (18:1/18:2) [1]	-13%	0.0014	0.0047
oleoyl-linoleoyl-glycerol (18:1/18:2) [2]	-12%	0.0044	0.0127
oxalate (ethanedioate)	-8%	0.0149	0.0353
palmitate (16:0)	41%	<0.0001	<0.0001
palmitoleate (16:1n7)	24%	0.0007	0.0027
palmitoleoylcarnitine (C16:1)	35%	0.0066	0.0177
palmitoleoyl-linoleoyl-glycerol (16:1/18:2) [1]	-29%	0.0005	0.0019
palmitoloelycholine	-16%	0.0042	0.0123
palmitoyl dihydrosphingomyelin (d18:0/16:0)	38%	<0.0001	<0.0001
palmitoyl sphingomyelin (d18:1/16:0)	12%	<0.0001	<0.0001
palmitoylcarnitine (C16)	20%	0.0007	0.0025
paraxanthine	13%	0.0036	0.0108
pentadecanoylcholine	40%	0.0029	0.0088
perfluorooctanoate (PFOA)	4%	0.0117	0.0285
phenylacetylcarnitine	91%	0.0188	0.0429
phosphate	7%	0.0001	0.0004
phytanate	450%	<0.0001	<0.0001
picolinate	53%	<0.0001	0.0001
pristanate	590%	<0.0001	<0.0001
propionylcarnitine (C3)	39%	0.0001	0.0004
pseudouridine	-7%	0.0002	0.0007
pyruvate	-27%	0.0008	0.0029
retinol (Vitamin A)	5%	0.0199	0.0448
ribonate	-17%	0.0024	0.0076
ribulonate/xylulonate	-12%	0.0074	0.0194
S-allylcysteine	-63%	0.0005	0.0020
sarcosine	48%	0.0001	0.0005
sebacate (C10-DC)	172%	0.0003	0.0013

serine	11%	0.0017	0.0055
S-methylcysteine sulfoxide	104%	<0.0001	<0.0001
S-methylmethionine	-80%	0.0003	0.0013
sphingomyelin (d17:1/14:0. d16:1/15:0)	31%	<0.0001	<0.0001
sphingomyelin (d17:1/16:0. d18:1/15:0. d16:1/17:0)	21%	<0.0001	<0.0001
sphingomyelin (d17:2/16:0. d18:2/15:0)	24%	<0.0001	<0.0001
sphingomyelin (d18:0/18:0. d19:0/17:0)	136%	<0.0001	<0.0001
sphingomyelin (d18:0/20:0. d16:0/22:0)	71%	<0.0001	<0.0001
sphingomyelin (d18:1/14:0. d16:1/16:0)	22%	<0.0001	<0.0001
sphingomyelin (d18:1/17:0. d17:1/18:0. d19:1/16:0)	37%	<0.0001	<0.0001
sphingomyelin (d18:1/18:1. d18:2/18:0)	29%	<0.0001	<0.0001
sphingomyelin (d18:1/19:0. d19:1/18:0)	28%	<0.0001	<0.0001
sphingomyelin (d18:1/20:2. d18:2/20:1. d16:1/22:2)	77%	0.0008	0.0029
sphingomyelin (d18:1/21:0. d17:1/22:0. d16:1/23:0)	18%	<0.0001	0.0002
sphingomyelin (d18:1/22:2. d18:2/22:1. d16:1/24:2)	20%	0.0003	0.0011
sphingomyelin (d18:1/25:0. d19:0/24:1. d20:1/23:0. d19:1/24:0)	46%	<0.0001	<0.0001
sphingomyelin (d18:2/14:0. d18:1/14:1)	18%	<0.0001	0.0001
sphingomyelin (d18:2/16:0. d18:1/16:1)	9%	0.0007	0.0027
sphingomyelin (d18:2/18:1)	61%	<0.0001	<0.0001
sphingomyelin (d18:2/21:0. d16:2/23:0)	40%	<0.0001	<0.0001
sphingomyelin (d18:2/23:0. d18:1/23:1. d17:1/24:1)	22%	<0.0001	<0.0001
sphingomyelin (d18:2/23:1)	36%	<0.0001	<0.0001
sphingomyelin (d18:2/24:2)	12%	0.0149	0.0353
stachydrine	-27%	0.0107	0.0265
stearate (18:0)	35%	<0.0001	0.0001
stearidonate (18:4n3)	46%	0.0007	0.0027
stearoyl sphingomyelin (d18:1/18:0)	31%	<0.0001	<0.0001
stearoylcarnitine (C18)	25%	0.0007	0.0027
suberate (C8-DC)	251%	<0.0001	<0.0001
suberoylcarnitine (C8-DC)	720%	<0.0001	<0.0001
succinylcarnitine (C4-DC)	-10%	0.0163	0.0381
sucrose	327%	0.0024	0.0077
sulfate	23%	<0.0001	<0.0001
tartronate (hydroxymalonate)	-14%	0.0053	0.0146
tauro-beta-muricholate	364%	0.0004	0.0015
taurochenodeoxycholate	87%	0.0108	0.0267
tetradecadienoate (14:2)	118%	<0.0001	<0.0001
tetradecanedioate (C14-DC)	197%	<0.0001	<0.0001
theophylline	38%	0.0016	0.0052
threonate	-6%	0.0180	0.0412
thymol sulfate	64%	0.0174	0.0403
tiglylcarnitine (C5:1-DC)	153%	<0.0001	<0.0001
tricosanoyl sphingomyelin (d18:1/23:0)	25%	<0.0001	<0.0001
trigonelline (N-methylnicotinate)	-31%	0.0135	0.0325
tyrosine	14%	0.0012	0.0040

urea	51%	<0.0001	<0.0001
valine	33%	<0.0001	<0.0001
vanillactate	-21%	0.0002	0.0010
xanthurenate	37%	0.0191	0.0430

ESM Table 5. Cardiorespiratory fitness and body composition at baseline and after the intervention period. Values are observed means ± SD. Estimates with 95% CI and *p* values for the effect compared to the control group, from linear mixed models.

	Baseline (Visit 1)	At study completion (Visit 3)	Estimate	95% CI	*p* value
	**Mean ± SD**	**Mean ± SD**			
**VO_2peak (ml kg^{-1} min^{-1})**					
Control	29.0 ± 4.3	28.5 ± 3.9			
EXam	28.5 ± 6.1	29.7 ± 6.2	1.3	0.5 to 2.0	**0.003**
EXpm	31.2 ± 4.4	33.4 ± 5.0	1.4	0.6 to 2.2	**0.001**
**Peak power output (W)**					
Control	193 ± 33	191 ± 34			
EXam	209 ± 65	220 ± 65	6.8	0.5 to 13.1	**0.04**
EXpm	208 ± 26	234 ± 29	14.4	8.1 to 20.7	**<0.001**
**Body mass (kg)**					
Control	94.5 ± 10.4	95.4 ± 9.8			
EXam	103.1 ± 16.1	102.1 ± 15.9	1.6	-1.9 to 5.2	0.36
EXpm	99.3 ± 8.1	98.6 ± 8.3	1.8	-1.8 to 5.3	0.32
**Waist circumference (cm)**					
Control	107.6 ± 5.9	105.9 ± 5.4			
EXam	110.7 ± 10.5	110.7 ± 10.2	1.2	-0.4 to 2.8	0.13
EXpm	109.1 ± 8.3	106.8 ± 8.7	-0.2	-1.6 to 1.3	0.84
**Waist-hip ratio**					
Control	0.99 ± 0.07	0.98 ± 0.07			
EXam	0.97 ± 0.05	0.97 ± 0.04	0.01	-0.01 to 0.02	0.37
EXpm	0.97 ± 0.06	0.95 ± 0.06	0.00	-0.01 to 0.1	0.55
**Fat mass (kg)**					
Control	31.3 ± 3.0	31.1 ± 3.2			
EXam	36.1 ± 7.0	35.2 ± 7.0	-0.29	-0.68 to 0.10	0.14
EXpm	32.9 ± 5.3	32.7 ± 5.4	-0.01	-0.39 to 0.38	0.98
**Fat free mass (kg)**					
Control	65.4 ± 9.0	64.7 ± 9.0			
EXam	67.2 ± 10.1	66.7 ± 9.6	0.13	-0.34 to 0.61	0.57
EXpm	66.7 ± 3.9	66.0 ± 3.8	0.07	-0.41 to 0.55	0.77
**Visceral fat mass (g)**					
Control	1969 ± 495	1906 ± 475			
EXam	2013 ± 1424	1826 ± 1382	-61	-122 to 0.4	0.05
EXpm	1479 ± 573	1356 ± 508	-0.34	-96 to 27	0.26

ESM Table 6. Fasting and postprandial circulating markers of metabolic health and blood pressure after five days of high-fat diet (HFD) and at study completion, according to group. Values are observed means ± SD. Estimates with 95% CI and *p* values for the interaction between time and group, from linear mixed models. Data were log or square root transformed when they had non-normal distribution (insulin, triacylglycerol, HOMA-IR, morning LDL). Estimates and 95% CIs for log-transformed variables are reported (in brackets). EXam = morning exercise, EXpm = evening exercise.

	**After 5 d high-fat diet**	**At study completion**	**Estimate**	**95% CI**	***p* value**
**MORNING (fasting)**					
**Glucose (mmol/l)**					
Control	4.8 ± 0.2	4.7 ± 0.3			
EXam	4.9 ± 0.5	4.8 ± 0.3	0.01	-0.09 to 0.12	0.85
EXpm	4.6 ± 0.2	4.4 ± 0.2	-0.12	-0.23 to -0.02	**0.02**
**Insulin (pmol/l)**					
Control	70 ± 35	59 ± 33			
EXam	74 ± 52	36 ± 19	(-0.46)	(-1.21 to 0.30)	0.22
EXpm	40 ± 24	24 ± 10	(-0.94)	(-1.69 to -0.19)	**0.02**
**HOMA-IR**					
Control	2.67 ± 1.7	1.58 ± 1.3			
EXam	2.33 ± 1.2	1.48 ± 0.8	(-0.09)	(-0.24 to 0.05)	0.20
EXpm	1.63 ± 1.2	1.15 ± 0.6	(-0.18)	(-0.33 to -0.04)	**0.01**
**Cholesterol (mmol/l)**					
Control	5.1 ± 0.8	4.9 ± 0.8			
EXam	5.1 ± 0.6	4.6 ± 0.4	-0.14	-0.35 to 0.07	0.19
EXpm	4.3 ± 0.6	3.7 ± 0.5	-0.38	-0.59 to -0.18	**<0.001**
**HDL (mmol/l)**					
Control	1.01 ± 0.2	0.99 ± 0.2			
EXam	1.02 ± 0.1	1.00 ± 0.1	0.02	-0.03 to 0.07	0.36
EXpm	1.10 ± 0.2	1.01 ± 0.1	-0.02	-0.07 to 0.03	0.37
**LDL (mmol/l)**					
Control	3.4 ± 0.7	3.3 ± 0.7			
EXam	3.3 ± 0.8	3.1 ± 0.4	(-0.01)	(-0.04 to 0.02)	0.61
EXpm	2.9 ± 0.5	2.4 ± 0.5	(-0.05)	(-0.08 to -0.02)	**0.001**
**Triacylglycerol (mmol/l)**					
Control	1.48 ± 0.6	1.35 ± 0.6			
EXam	1.44 ± 0.8	0.97 ± 0.3	(-0.04)	(-0.10 to 0.01)	0.09
EXpm	0.83 ± 0.1	0.68 ± 0.1	(-0.09)	(-0.14 to -0.03)	**0.002**
**Systolic blood pressure (mm Hg)**					
Control	129 ± 9	129 ± 11			
EXam	125 ± 11	124 ± 4	-1.1	-4.3 to 2.1	0.50
EXpm	122 ± 10	127 ± 6	-0.9	-4.5 to 2.0	0.42
**Diastolic blood pressure (mm Hg)**					
Control	84 ± 4	87 ± 9			
EXam	80 ± 5	80 ± 2	-1.6	-3.6 to 0.4	0.12
EXpm	82 ± 6	83 ± 4	-1.4	-3.4 to 0.6	0.17
**EVENING (postprandial)**					
**Glucose (mmol/l)**					
Control	5.2 ± 0.4	5.1 ± 0.5			
EXam	4.9 ± 0.4	4.7 ± 0.5	-0.11	-0.26 to 0.03	0.12
EXpm	5.2 ± 0.8	4.6 ± 0.3	-0.12	-0.26 to o.03	0.11
**Insulin (pmol/l)**					
Control	432 ± 252	283 ± 145			
EXam	189 ± 130	129 ± 65	(-2.13)	(-3.67 to -0.60)	**0.008**
EXpm	230 ± 161	139 ± 99	(-1.89)	(-3.42 to 0.35)	**0.02**

**Cholesterol (mmol/l)**					
Control	5.4 ± 0.8	5.1 ± 0.7			
EXam	5.3 ± 0.8	4.7 ± 0.6	-0.15	-0.37 to 0.07	0.18
EXpm	4.5 ± 0.7	3.9 ± 0.5	-0.37	-0.59 to -0.15	**0.002**
**Triacylglycerol (mmol/l)**					
Control	2.97 ± 1.2	2.29 ± 0.6			
EXam	2.09 ± 0.7	1.78 ± 0.9	(-0.05)	(-0.11 to 0.01)	0.10
EXpm	1.73 ± 0.7	1.20 ± 0.3	(-0.09)	(-0.15 to -0.04)	**0.003**
**HDL (mmol/l)**					
Control	1.02 ± 0.1	1.01 ± 0.1			
EXam	1.05 ± 0.1	1.01 ± 0.1	0.00	-0.04 to 0.05	0.90
EXpm	1.12 ± 0.2	1.07 ± 0.2	0.01	-0.04 to 0.05	0.73
**LDL (mmol/l)**					
Control	3.1 ± 0.9	3.0 ± 0.8			
EXam	3.2 ± 0.7	2.9 ± 0.4	-0.10	-0.30 to 0.11	0.36
EXpm	2.6 ± 0.5	2.3 ± 0.4	-0.25	-0.45 to -0.04	**0.02**
**Systolic blood pressure (mm Hg)**					
Control	132 ± 5	132 ± 9			
EXam	133 ± 9	129 ± 8	-0.9	-3.4 to 1.6	0.47
EXpm	131 ± 6	132 ± 4	-0.1	-2.6 to 2.4	0.94
**Diastolic blood pressure (mm Hg)**					
Control	86 ± 7	88 ± 6			
EXam	84 ± 6	83 ± 6	-1.6	-3.6 to 0.4	0.11
EXpm	86 ± 6	84 ± 5	-1.3	-3.3 to 0.7	0.19

ESM Table 7. Fasting and postprandial circulating markers of metabolic health and blood pressure after five days of high-fat diet (HFD) and at study completion, according to group. Values are observed means ± SD. Estimates with 95% CI and *p* values for the effect of evening exercise (EXpm) compared to morning exercise (EXam), from linear mixed models. Data were log or square root transformed when they had non-normal distribution (insulin, triacylglycerol, HOMA-IR, morning LDL). Estimates and 95% CIs for log-transformed variables are reported (in brackets).

	**After 5 d high-fat diet**	**At study completion**	**Estimate**	**95% CI**	***p* value**
**MORNING (fasting)**					
**Glucose (mmol/l)**					
EXam	4.9 ± 0.5	4.8 ± 0.3			
EXpm	4.6 ± 0.2	4.4 ± 0.2	-0.15	-0.24 to -0.03	**0.02**
**Insulin (pmol/l)**					
EXam	74 ± 52	36 ± 19			
EXpm	40 ± 24	24 ± 10	(-0.48)	(-1.23 to -0.27)	0.20
**HOMA-IR**					
EXam	2.33 ± 1.2	1.48 ± 0.8			
EXpm	1.63 ± 1.2	1.15 ± 0.6	(-0.09)	(-0.24 to 0.06)	0.22
**Cholesterol (mmol/l)**					
EXam	5.1 ± 0.6	4.6 ± 0.4			
EXpm	4.3 ± 0.6	3.7 ± 0.5	-0.25	-0.46 to -0.04	**0.02**
**HDL (mmol/l)**					
EXam	1.02 ± 0.1	1.00 ± 0.1			
EXpm	1.10 ± 0.2	1.01 ± 0.1	-0.04	-0.09 to 0.00	0.08
**LDL (mmol/l)**					
EXam	3.3 ± 0.8	3.1 ± 0.4			
EXpm	2.9 ± 0.5	2.4 ± 0.5	(-0.04)	(-0.07 to -0.01)	**0.006**
**Triacylglycerol (mmol/l)**					
EXam	1.44 ± 0.8	0.97 ± 0.3			
EXpm	0.83 ± 0.1	0.68 ± 0.1	(-0.04)	(-0.09 to 0.01)	0.12
**Systolic blood pressure (mm Hg)**					
EXam	125 ± 11	124 ± 4			
EXpm	122 ± 10	127 ± 6	-0.2	-3.5 to 2.0	0.89
**Diastolic blood pressure (mm Hg)**					
EXam	80 ± 5	80 ± 2			
EXpm	82 ± 6	83 ± 4	-0.2	-1.9 to 2.2	0.17
**EVENING (postprandial)**					
**Glucose (mmol/l)**					
EXam	4.9 ± 0.4	4.7 ± 0.5			
EXpm	5.2 ± 0.8	4.6 ± 0.3	-0.003	-0.15 to 0.14	0.96
**Insulin (pmol/l)**					
EXam	189 ± 130	129 ± 65			
EXpm	230 ± 161	139 ± 99	(0.25)	(-1.29 to 1.78)	0.74
**Cholesterol (mmol/l)**					
EXam	5.3 ± 0.8	4.7 ± 0.6			
EXpm	4.5 ± 0.7	3.9 ± 0.5	-0.21	-0.44 to 0.01	0.06
**Triacylglycerol (mmol/l)**					
EXam	2.09 ± 0.7	1.78 ± 0.9			
EXpm	1.73 ± 0.7	1.20 ± 0.3	(-0.05)	(-0.10 to 0.01)	0.12
**HDL (mmol/l)**					
EXam	1.05 ± 0.1	1.01 ± 0.1			
EXpm	1.12 ± 0.2	1.07 ± 0.2	0.004	-0.04 to 0.05	0.84
**LDL (mmol/l)**					
EXam	3.2 ± 0.7	2.9 ± 0.4			

EXpm	2.6 ± 0.5	2.3 ± 0.4	-0.15	-0.36 to 0.06	0.15
**Systolic blood pressure (mm Hg)**					
EXam	133 ± 9	129 ± 8			
EXpm	131 ± 6	132 ± 4	0.8	-1.7 to 3.3	0.52
**Diastolic blood pressure (mm Hg)**					
EXam	84 ± 6	83 ± 6			
EXpm	86 ± 6	84 ± 5	-0.3	-1.7 to 2.3	0.77

ESM Table 8. Metabolites differently changed in morning samples after evening exercise compared to no exercise, using linear mixed model.

	Change (%) after morning exercise (EXam)	Change (%) after evening exercise (EXpm)	Change (%) after no exercise (CON)	*q* value between-group difference between EXpm and CON
androsterone sulfate	12.2	33.4	-9.9	<0.0001
epiandrosterone sulfate	10.9	40.5	-6.2	<0.0001
5alpha-androstan-3alpha,17beta-diol monosulfate (1)	8.8	38.4	-15.0	<0.0001
andro steroid monosulfate C19H28O6S (1)	46.8	64.4	-4.5	<0.0001
16a-hydroxy DHEA 3-sulfate	34.8	64.2	-1.6	<0.0001
carnitine	-6.8	-10.5	3.6	<0.0001
1-oleoyl-2-docosahexaenoyl-GPC (18:1/22:6)	-6.7	-17.5	3.3	0.001
5alpha-androstan-3beta.17beta-diol monosulfate (2)	17.8	45.9	-16.4	0.001
androstenediol (3beta.17beta) disulfate (2)	11.3	19.2	-0.6	0.002
1-stearoyl-2-oleoyl-GPC (18:0/18:1)	-10.2	-16.7	-1.8	0.002
1-palmitoleoyl-2-linolenoyl-GPC (16:1/18:3)	-20.0	-38.2	-1.8	0.002
tryptophan	-5.6	-6.4	3.1	0.003
dehydroisoandrosterone sulfate (DHEA-S)	-1.4	18.0	-4.7	0.004
alpha-hydroxyisovalerate	56.8	64.5	-6.3	0.004
gamma-glutamylglycine	1.4	-9.2	16.7	0.005
1-linoleoylglycerol (18:2)	-20.2	11.2	-29.0	0.007
pyroglutamine	-8.8	-17.4	4.0	0.007
N-acetylthreonine	-7.6	-9.7	3.3	0.007
2-hydroxy-3-methylvalerate	19.4	62.5	-5.8	0.007
hippurate	-11.3	156.1	44.8	0.007
acetoacetate	130.8	222.4	24.9	0.007
3-hydroxybutyroylglycine	157.4	176.5	11.8	0.008
1-palmitoyl-2-dihomo-linolenoyl-GPC (16:0/20:3n3 or 6)	-11.5	-18.7	-4.0	0.008
androstenediol (3alpha, 17alpha) monosulfate (3)	-4.3	11.0	-4.5	0.008
gamma-glutamylserine	12.9	-4.5	19.1	0.008
suberate (C8-DC)	171.4	291.1	35.6	0.010
2-hydroxybutyrate/2-hydroxyisobutyrate	47.0	71.4	-3.8	0.010
azelate (C9-DC)	109.7	341.9	128.8	0.010
sphingomyelin (d17:1/14:0, d16:1/15:0)	-19.1	-20.9	1.3	0.012
androstenediol (3beta,17beta) monosulfate (1)	-1.1	18.9	-11.0	0.012
beta-hydroxybutyrate (βOHB)	184.7	379.2	25.5	0.015
dihydroorotate	0.9	-5.1	15.3	0.015
isobutyrylglycine	57.5	44.2	-26.7	0.022
2-methylcitrate/homocitrate	6.2	28.3	-5.5	0.023
succinylcarnitine (C4-DC)	-15.0	-37.4	-11.2	0.024
sphingomyelin (d18:2/14:0, d18:1/14:1)	-14.9	-20.2	-2.3	0.024
indolelactate	22.6	30.1	4.0	0.024
androstenediol (3beta,17beta) disulfate (1)	4.5	6.5	-15.8	0.026
3-hydroxysebacate	44.6	89.5	-17.9	0.028
3-hydroxybutyrylcarnitine (1)	145.1	187.6	16.5	0.029
5alpha-androstan-3beta,17beta-diol disulfate	2.9	3.3	-13.4	0.029
6-bromotryptophan	1.9	-22.1	-0.3	0.029
propionylcarnitine (C3)	-23.7	-29.2	-6.7	0.030
pantothenate	6.7	12.2	-5.9	0.032
hexanoylglycine	119.4	138.6	113.9	0.033
citrate	34.1	52.0	10.9	0.041
mannonate	1.2	-4.2	14.0	0.046
tyrosine	-4.1	-9.7	2.1	0.046

myristoyl dihydrosphingomyelin (d18:0/14:0)	-21.1	-17.8	-3.7	0.046
N-acetylglycine	53.5	37.6	12.5	0.046
aconitate [cis or trans]	28.4	57.2	18.9	0.046
ceramide (d16:1/24:1, d18:1/22:1)	-12.9	-20.0	3.0	0.046
creatine	-17.9	-26.7	-3.2	0.046
4-methylcatechol sulfate	-3.6	81.9	7.7	0.046
1-myristoyl-2-arachidonoyl-GPC (14:0/20:4)	-29.9	-37.5	-12.6	0.046
5alpha-androstan-3beta,17alpha-diol disulfate	8.5	32.3	-2.1	0.048

ESM Table 9. Metabolites differently changed in evening samples with evening exercise compared to no exercise, using linear mixed model.

	Change (%) after morning exercise (EXam)	Change (%) after evening exercise (EXpm)	Change (%) after no exercise (CON)	*q* value between-group difference between EXpm and CON
androsterone sulfate	13.10	41.980	-7.239	<0.0001
epiandrosterone sulfate	15.16	48.003	-3.343	0.0004
5alpha-androstan-3alpha,17beta-diol monosulfate (1)	15.35	39.303	-9.318	0.0017
1-palmitoyl-2-dihomo-linolenoyl-GPC (16:0/20:3n3 or 6)	-10.85	-14.717	-1.195	0.0080
5alpha-androstan-3beta,17beta-diol monosulfate (2)	20.74	50.269	-11.331	0.0117
betaine	-2.82	4.880	-11.805	0.0129
alpha-hydroxyisovalerate	31.02	55.338	-14.765	0.0232
